# The Route from the Folded to the Amyloid State: Exploring the Potential Energy Surface of a Drug‐Like Miniprotein

**DOI:** 10.1002/chem.201903826

**Published:** 2019-12-27

**Authors:** Nóra Taricska, Dániel Horváth, Dóra K. Menyhárd, Hanna Ákontz‐Kiss, Masahiro Noji, Masatomo So, Yuji Goto, Toshimichi Fujiwara, András Perczel

**Affiliations:** ^1^ Laboratory of Structural Chemistry and Biology & MTA-ELTE Protein Modeling Research Group Eötvös Loránd University Pázmány Péter sétány 1A 1117 Budapest Hungary; ^2^ Institute for Protein Research Osaka University 3-2 Yamadaoka Suita, Osaka 565-0871 Japan

**Keywords:** aggregation, amyloid beta-peptides, protein folding, protein modeling

## Abstract

The amyloid formation of the folded segment of a variant of Exenatide (a marketed drug for type‐2 diabetes mellitus) was studied by electronic circular dichroism (ECD) and NMR spectroscopy. We found that the optimum temperature for E5 protein amyloidosis coincides with body temperature and requires well below physiological salt concentration. Decomposition of the ECD spectra and its barycentric representation on the folded‐unfolded‐amyloid potential energy surface allowed us to monitor the full range of molecular transformation of amyloidogenesis. We identified points of no return (e.g.; *T*=37 °C, pH 4.1, *c*_E5_=250 μm, *c*_NaCl_=50 mm, *t*>4–6 h) that will inevitably gravitate into the amyloid state. The strong B‐type far ultraviolet (FUV)‐ECD spectra and an unexpectedly strong near ultraviolet (NUV)‐ECD signal (*Θ*_≈275–285__nm_) indicate that the amyloid phase of E5 is built from monomers of quasi*‐*elongated backbone structure (*φ*≈−145°, *ψ*≈+145°) with strong interstrand Tyr↔Trp interaction. Misfolded intermediates and the buildup of “toxic” early‐stage oligomers leading to self‐association were identified and monitored as a function of time. Results indicate that the amyloid transition is triggered by subtle misfolding of the α‐helix, exposing aromatic and hydrophobic side chains that may provide the first centers for an intermolecular reorganization. These initial clusters provide the spatial closeness and sufficient time for a transition to the β‐structured amyloid nucleus, thus the process follows a nucleated growth mechanism.

## Graphical abstract

**Centers of reorganization**: The amyloid formation of the folded segment of a variant of Exenatide (a marketed drug for type‐2 diabetes mellitus) was studied by electronic circular dichroism (ECD) and NMR spectroscopy. Results indicate that the amyloid transition is triggered by subtle misfolding of the α‐helix, exposing aromatic and hydrophobic side chains that may provide the first centers for an intermolecular reorganization.

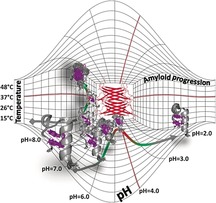

## Introduction

Aggregation of proteins and peptide segments into amyloid fibrils have been studied intensively over the past decades since the process was shown to be associated with, or even trigger,^1^, ^2^, ^3^ such illnesses as Alzheimer's disease, type‐2 diabetes mellitus, rheumatoid arthritis, or haemodialysis ass. amyloidosis.^4^ From the pioneering work on lysozyme^5^ and Aβ(1–42),^6^, ^7^, ^8^ the amyloid state of several misfolded proteins (e.g., β2‐microglobulin,^9^ crystallin,^10^ tau protein,^11^, ^12^, ^13^ the glucagon peptide hormone,^14^ and insulin^15^ among others) were characterized. The general topology of such aggregates consists of protein segments adopting an extended backbone, interacting through β‐edges. The association between the β‐sheets thus formed is compact and specific; in most cases it excludes water molecules, leading to the formation of tightly stacked, “dry‐zipper” nanostructures.^16^, ^17^, ^18^ The state‐of‐the‐art TEM, SAXS, cryo‐EM and ssNMR techniques now allow full characterization of the aggregated end‐state;^19^, ^20^ however, much less is known about the specific molecular species that evolve during the process, especially in the early stages, which concern the formation of still soluble but oligomeric assemblies that are the most toxic^1^, ^2^, ^3^, ^21^, ^22^ and also represent the stage at which amyloidosis can still be reversed.^23^

The progress of self‐association can often be followed by ThT fluorescence and DLS—best in combination^24^—reporting the accumulation of cross‐β‐backbone (above a minimum size of ca. 10 nm or 4–6 aligned strands^25^) and the size distribution of the species present in the solution, respectively. However, to gain atomistic detail, molecular spectroscopy needs to be applied such as CD, IR or NMR spectroscopy.^26^, ^27^ In fact, CD spectroscopy can be used to monitor a full range of molecular transformations accompanying amyloidogenesis if the secondary structure content of the folded, intermediate, and amyloid states are distinct. There are notable examples that satisfy this description; namely, helical peptide hormones such as amylin or glucagon and a considerable number of peptide therapeutics^28^ and since over 60 % of all protein‐protein interfaces—typical targets of drug design—also constitute helices,^29^ their number will most likely just increase.

**Figure 1:**
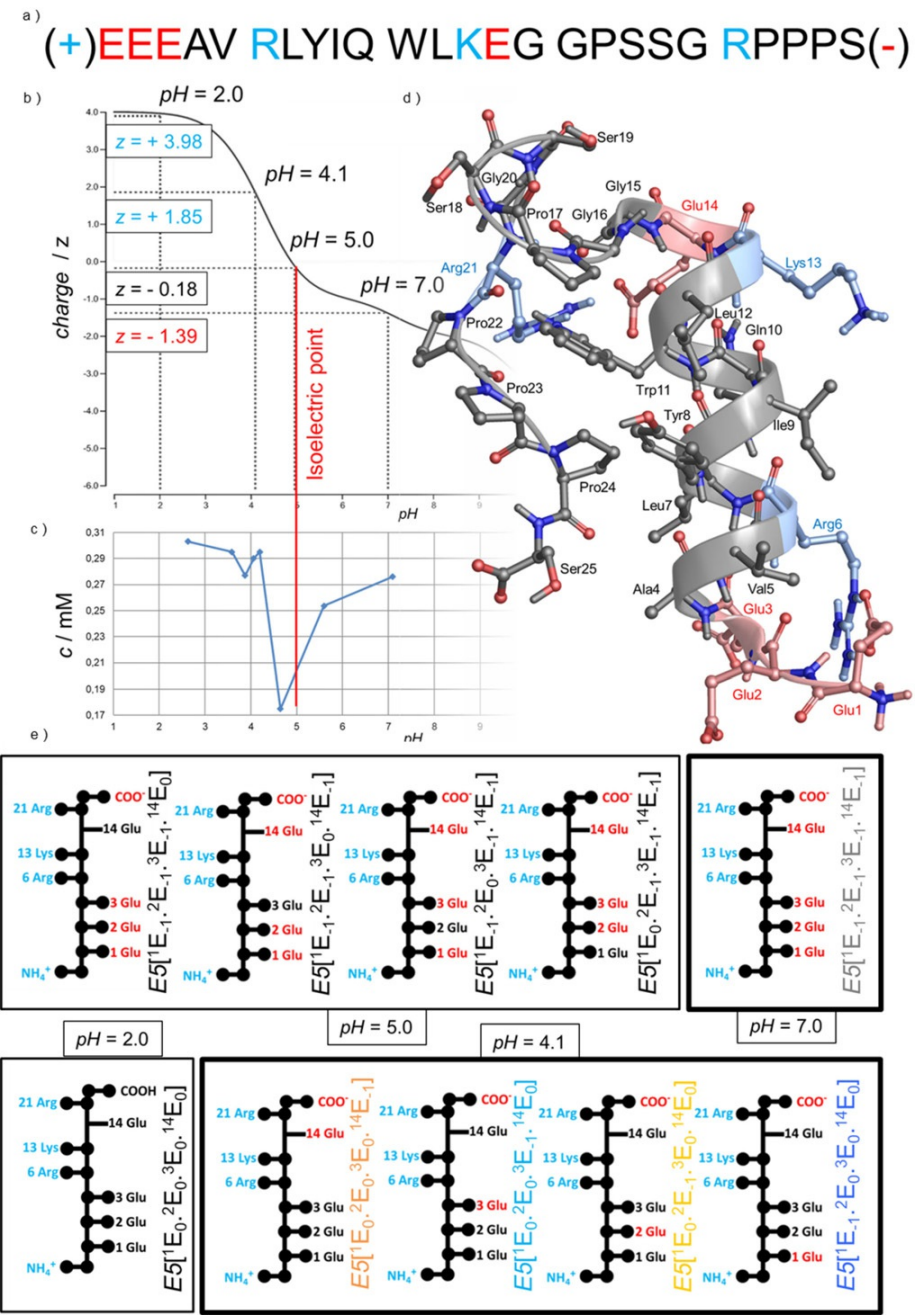
a) Primary sequence of E5 with residues colored by their charges of pH 7.0: negatively charged amino acid, positively charged amino acid, and neutral amino acid are highlighted with red, blue, and black, respectively. b) Overall charge (*z*) of E5 as a function of the pH calculated by Prot pi (http://www.protpi.ch). c) The solubility of E5 as a function of the pH (at 25 °C) shows that near the isoelectric point reversible precipitation occurs. d) Folded structure of E5 (4 °C & pH 7.0) determined by NMR analysis, with its four basic groups (highlighted blue), and five acidic groups (highlighted red) with the following p*K*_a_ values: Ser_C‐terminal_: 2.2, Glu_side chain_: 4.3, Glu_N‐terminal_: 9.1, Lys_side chain_: 10.8, and Arg_side chain_: 12.5. e) The major microstates with their labeling are depicted schematically at four different pH values of interest, with side chains colored by charge: neutral (black), negative (red) and positive (blue).

Here we present the amyloid formation pathway of a variant of Exenatide, a marketed drug for type‐2 diabetes mellitus^30^ that also contains a well‐folded α‐helix. We have discovered that this 25 residue long segment (E5: EEEAVRLYIQWLKEGGPSSGRPPPS) (Figure 1), comprising the entire interface needed for GLP‐1 receptor binding,^31^ can be turned into amyloid in a controlled, fully reproducible and tunable manner within a large range of protein concentrations (80 μm<*c*_protein_<800 μm) at physiologically relevant temperatures. Therefore, understanding the molecular details of the amyloidosis of E5 and mapping its conditions is highly relevant to any optimization efforts targeting Exenatide. In addition, E5 is an ideal model to study the amyloid transition of folded proteins and helix‐containing peptides. Beside its helical stretch, E5 contains a β‐turn, a polyproline‐II helix and a hydrophobic center with a buried Trp, thus has a protein‐like build‐up and also folds quite similarly to a typical globular protein.^32^

As E5 is small, both its chemical synthesis on a resin and bacterial expression in a fusion system is straightforward.^33^ Given that its folded state is partially helical, its transition toward the amyloid phase results in a significant change in secondary structure content, which is easy to monitor by electronic circular dichroism (ECD) spectroscopy. Furthermore, E5 has an interacting Trp/Tyr residue pair within its hydrophobic core, enabling folding and refolding of the protein to be tracked by near ultraviolet (NUV)‐ in addition to far ultraviolet (FUV)‐ECD. We also found the process to be quenchable; the amyloid formation can be suspended at any time by dropping the temperature, and restarted by a subsequent temperature rise. Furthermore, the moderate size of E5 allows detailed structure characterization both by NMR and modeling techniques.

We probed various regions of the *f*(*T*,pH,*c*_protein_,*c*_ion_,*t*) potential energy hypersurface of E5 by acquiring quantitative NUV‐ and FUV‐ECD chiroptical information and NMR data complemented by MD simulations to pinpoint the reaction path that leads from the fully folded‐ to the amyloid‐state. Based on these results, we were able to propose a mechanism that resembles that of well‐folded proteins but relies on special features of the miniprotein. The methodology presented here gains significance because the amyloid state of E5 is ThT silent and thus presents an approach for dealing with such cases as well.

## Results and Discussion

We have shown previously^34^ that high concentrations (*c*≈10–30 mm) of E5 trigger self‐association, accompanied by an α‐helix to antiparallel β‐sheet structural transition. Here, we set out to identify optimum conditions of aggregation, structurally characterizing key states along this route at a physiologically more relevant concentration range using NMR, NUV‐ and FUV‐ECD data to pinpoint misfolded structures of the reaction path, and electron microscopy to confirm the emerging amyloid fibrils.

**Figure 2:**
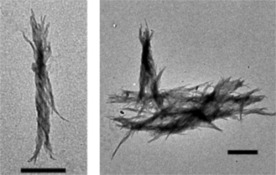
TEM images of E5 amyloid fibrils. Typical twisted amyloid fibrils were observed. Most fibrils were bundled and formed supercoiled structure. Scale bars: 500 nm.

To locate roughly and effectively those conditions that enable the amyloid formation, the HANABI system^35^ was applied. The 96 well plates were set up with the following boundaries: 2<pH<7, 80 μm<*c*_E5_<800 μm and 0 mm<*c*_NaCl_<100 mm at *T*=37 °C. ECD measurements were carried out after 90 h, with sonication cycle turned “on” for 1 min and repeated every 10 min (power level: 700 W and frequency: 25 kHz). FUV‐ECD measurements showed that amyloid formation is more effective in the presence than in the absence of salt (*c*_NaCl_=100 mm), with the preferred protein concentration ranging from 80 to 160 μm, while the optimal pH is near 4.0. The amyloid thus produced showed a typical twisted fibril structure (Figure 2).

The formation of these fibrils could not be followed by ThT fluorescence and thus ECD spectra were recorded for each of the 96 wells. Based on the results, additional experiments were designed to identify parameters of amyloid formation separately, to pinpoint their significance and specific molecular consequences.

### Optimum conditions located for E5 amyloid formation: the temperature‐scan

**Figure 3:**
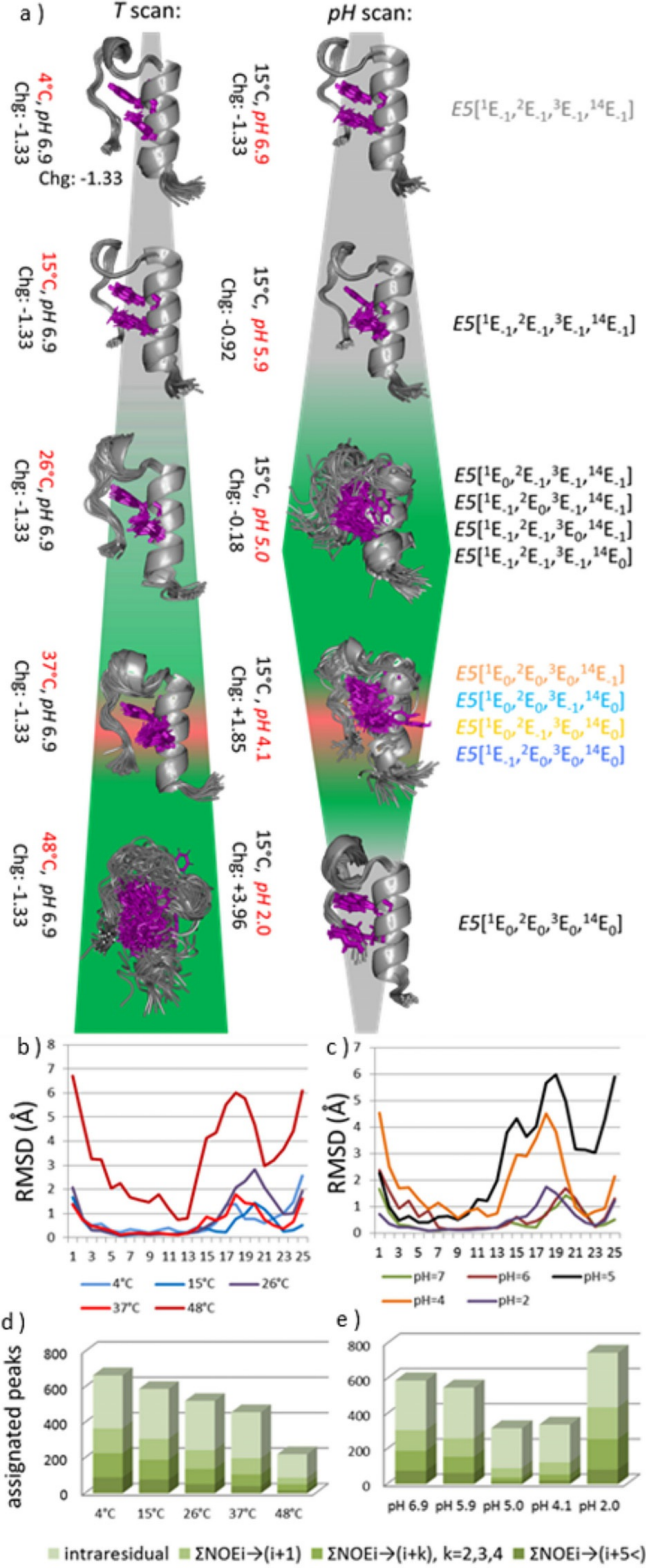
a) Superimposed best 50 NMR structures of E5 at different pH values and temperatures (conditions and the net charges explicitly shown for each structure ensemble). The background color indicates the fold compactness: green**—**unfolded, gray**—**folded, red**—**misfolded leading to amyloid formation. Aromatic side chains of the key Y^8^ and W^11^ are highlighted magenta. The basic (N‐terminal; R^6^, K^13^, R^21^) moieties are protonated, the C‐terminal (S^25^) deprotonated, whereas the protonation microstates of the Glu residues are shown at the right side at each pH. All atoms backbone RMSD (Å) as a function of the primary sequence of E5 at b) different temperatures (°C) and c) different pH values are plotted. The total number of NOEs assigned for E5: ΣNOE^*i*→(*i***+**1)^, ΣNOE^*i*→(*i*+*k*)^ where *k*=2,3,4, ΣNOE^*i*→(*i*+5<)^ are reported as d) the temperature was scanned at pH 6.9 and e) as pH was scanned at *T*=15 °C.

NMR (2D homonuclear) measurements were carried out to determine the structure of E5 varying the temperatures between 4 and 48 °C (at pH 7, *c*_protein_≈0.8 mm, *c*_ion_≈0 mm) leading to the primary conclusion that as temperature increases, partial unfolding occurs without amyloid formation. We found that although NMR frequencies shift with rising *T*, line broadening only takes place above 37 °C, indicating that considerable unfolding occurs only at 48 °C (Figure S1 in the Supporting Information). The unsynchronized local backbone fluctuation of the folded F‐state enhances as *T* increases. Furthermore, we have determined the most *T‐*sensitive residues of E5 for which the presence of hidden intermediate(s) (I‐state(s))^36^, ^37^ was revealed. The thermal unfolding of the protein backbone shows a nonlinear *T* dependence for Leu^7^, Ile^9^, and Lys^13^ and, to a lesser extent, for Ala^4^, Val^5^, and Tyr^8^, indicating an enhanced presence of transient conformers at higher temperatures for the inner helix of E5 (Figure 1, Figure S2). 3D structure elucidations were also completed by acquiring a large number of NOE distance‐restrains (Figure 3 and Table S1). Although the total number of restrains drops as *T* increases, 666 (4 °C)→221 (48 °C), the latter number of NOEs are still sufficient to establish the overall 3D‐fold of E5 even at 48 °C (especially since 16 out of 221 are key long‐range restraints) (Figure 3 e and Table S1). Inter‐residue NOEs associated with Y^8^ and W^11^ (Figure S3) enabled us not only to determine the overall molecular scaffold but also the relative orientation of the two aromatic side chains. At 37 °C, 458 distance restraints in total, among which 40 long range ones (*i*→(*i*+5<)) were assigned, affording a single time‐average and compact 3D structure for E5. While the central α‐helix is tightly folded at this temperature (RMSD of the backbone heavy atoms is 0.64 Å within the 50 best‐fit structures) (Table S1), as *T* increases the unfolding of the ‐P^17^SSGRP^22^‐ segment was detected. This segment is the least structured area within the folded scaffold even at 4 °C, where only sequential NOEs could be measured **(**Figure 3 c). Nevertheless, the large number of NOEs associated with Y^8^ and W^11^ residues and the ‐P^22^PP^24^‐ segment ensures their concerted motion, signaling that the hydrophobic core remains well‐structured at 37 °C and below (Figure S3).

Due to exchange phenomena, NMR spectroscopy cannot be used to provide precise structural information at high *T*, therefore complementary FUV‐ECD measurements were carried out to follow the transition of the backbone fold up to 85 °C. The recorded spectra were deconvoluted using the CCA+ protocol as mixtures of folded (F‐) and unfolded (U‐) forms.^38^, ^39^, ^40^ At lower temperatures, in line with the NMR data, the folded fraction is dominant (F content goes from 100 to 80 % as *T* increases from 5 to 35 °C), a 50:50 % mixture is reached near 60 °C, while at 85 °C the spectrum indicates a 70 % unfolded content (Figure S4).

### The pH‐scan

2D‐NOESY driven 3D structure elucidation was completed (*c*_E5_ ≈500–1500 μm, *c*_NaCl_ <10 mm) at various pH values (6.9, 5.9, 5.0, 4.1, 2.0) at *T*=15 °C (Figure 3 b) (The poorer signal to noise ratio meant that longer measurement time was required at pH 5.0 and 4.1). The solubility of E5 drops close to its isoelectric point (pH 4.8, Figure 1) where unspecific and reversible precipitation was observed. As side chain protonation pattern varies with pH, H‐bonds and other weak interactions also change. E5 contains a protonated N‐terminal (p*K*_a_=9.1) plus three basic residues, Arg^6^ (p*K*_a_=12.5), Lys^13^ (p*K*_a_=10.8) and Arg^21^ (p*K*_a_=12.5), with an acidic *C‐*terminal (p*K*_a_=2.2) and four acidic glutamines (Glu^1^, Glu^2^, Glu^3^ and Glu^14^) (p*K*_a_ ≈4.25) (the listed p*K*_a_ values are nominal values that strongly depend on backbone conformation). The net charge of E5 is predicted to be positive at pH values smaller than 5 (Figure 1). At pH 7.0, two salt bridges may contribute to the stabilization of the 3D‐fold, those of Glu^1^(−)↔Arg^6^(+) and Glu^14^(−)↔Arg^21^(+). But as pH decreases, Glu(s) get partly (or completely) protonated and thus salt bridges weaken and 3D‐fold compactness loosens. Accordingly, at pH 6.9, 5.9, and 5.0, the measured 3D structures of E5 are similar (Figure 3), although conformational heterogeneity increases considerably (demonstrated by the reduction of the total number of assigned restrains from 666 to 319; Figure 3 f and Table S1). Thus, the Trp‐cage fold holds, though backbone heavy atom RMSD of the 50 best structures increases significantly: RMSD (pH 6.9, *T=*15 °C)=0.73 Å → RMSD (pH 5.0, *T=*15 °C)=2.56 Å (Table S1). We found that the total number of NOESY cross‐peaks between R^21^ and W^11^ residues is a reliable measure of the Trp‐cage fold compactness (Figure S3): 11 and 10 such peaks were assigned at pH 6.9 and 2.0, but only 3 at 5>pH*>*4 (*T*=15 °C). Moreover, RMSD changes show that the α‐helix becomes partly unfolded, as the pH gets closer to 4.1 (Figure 3 d). The structure loosening effect of the pH drop is the most pronounced between residues 13 and 21: the 3_10_‐helix (‐G^15^GPSSG^20^‐) tends to unwind, exposing both the Y^8^ and W^11^ core residues to external water molecules (Figure 3). Interestingly, moving beyond the isoelectric point, ordering of the ensembles takes place and the original F‐state reappeared at pH 2.0. At pH 6.9, 77 long‐range NOE^*i*→(*i*+5<)^ restraints were assigned, while at pH 2.0 in total 84 NOEs^*i*→(*i*+5<)^ were found. This is rather unexpected since the overall charge, as well as the local charge distribution of E5 is indeed different at the above two pH values. Meanwhile, in between, at pH 4.9 only 22 NOEs of this kind were recorded (Figure 3 b). In conclusion, the pH‐scan shows that the basic topological features of the Trp‐cage fold of E5 (and other analogues^41^) are preserved at pH 7.0 and 2.0, but weakened near the isoelectric point, where I‐states of full refolding potential or of amyloidogenic misfolding capacity could be present simultaneously (Figure S5).

### The effect of stirring

At pH 4.1 and *T*=37 °C, in the absence of stirring or sonication, the gradual decay of the far‐UV ECD spectrum intensities of E5 was detected, which is indicative of self‐association leading to the loss of monomeric form or weakly bound low molecular weight associates in the solution (Figure S6). Stirring, however, greatly speeds up both the nucleation and fibril maturation stages of amyloidogenesis. This affects aggregation by increasing fragmentation and hence the number of free ends to support further fibril growth and possibly by increasing the number of collisions occurring between monomers and/or small oligomeric clusters.^42^, ^43^

**Figure 4:**
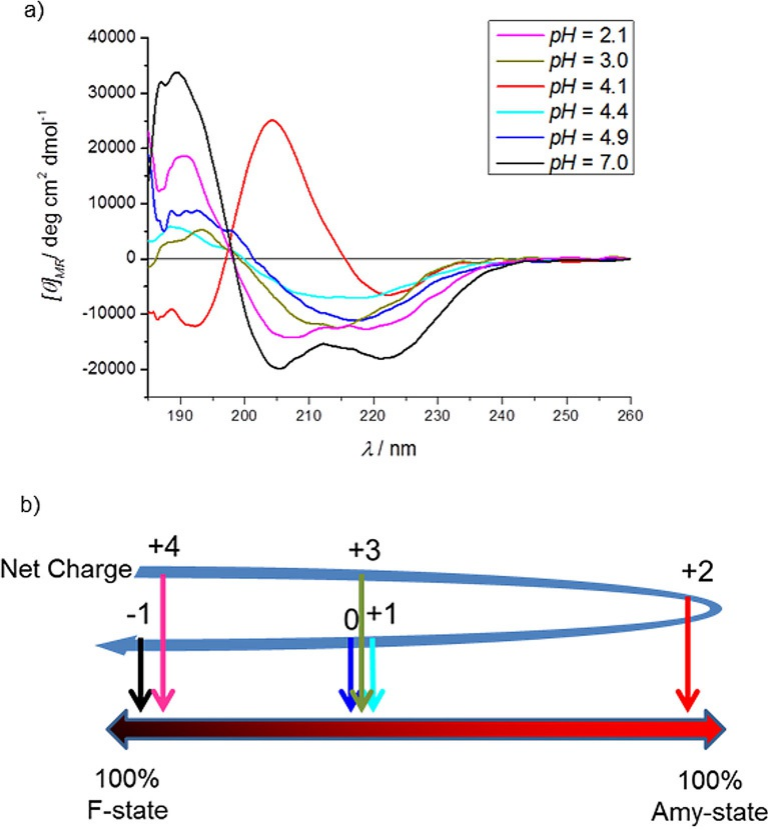
a) FUV‐ECD spectra of E5 recorded after 51 h of incubation time at constant stirring (*c*_E5_=250 μm, *c*_NaCl_=50 mm, *T*=37 °C). If ΣQ_E5_^pH 4.1^=+2, then a B‐type ECD is detected, which is indicative of a β**‐**sheet backbone structure. However, if pH 7.0 (ΣQ_E5_^pH 7.0^=−1) or pH 2.1 (ΣQ_E5_^pH 2.1^=+4.0) E5 remains folded (predominantly α**‐**helical). b) Spectral deconvolution enables the approximate [F] and [Amyloid] ratios to be determined as a function of the net molecular charge: ΣQ_E5_.

Stirring the solution of E5 for 51 h (pH 4.1) resulted in a strong B‐type FUV‐ECD spectrum, indicating a predominantly β‐pleated backbone structure.^44^, ^45^ When setting the pH to 4.9, 4.4, and 3.0, “mixed” ECD spectra were recorded (B/C‐type) even after 51 h (Figure 4) signaling that, in solution, both helical and β‐stranded structures are present, and amyloid formation is less complete. At pH 7 and pH 2, pure C‐type ECD spectra were measured (even when stirring), indicating that only α‐helical backbone structures are in solution and thus, these pH values prevent amyloid formation. To quantify the extent of the transition from the folded toward the amyloid‐like phase, deconvolution of a large collection of ECD spectra were carried out (including *T‐*dependent ECD curves of E5 and E0 as unfolded model miniprotein^37^). In this way, we obtained three pure component spectra: A C‐type for α‐helical conformation, a U‐type corresponding to the unstructured and a B‐type signaling β‐stranded backbone conformation. More interestingly, a similar NUV‐ECD spectrum analysis (see below) shows that this transition is more complex than a simple α‐ to β‐backbone conformational shift. Complemented with dynamic light scattering measurement data, we conclude that these NUV‐ECD spectral changes are associated with a gradual amyloid formation.

**Figure 5:**
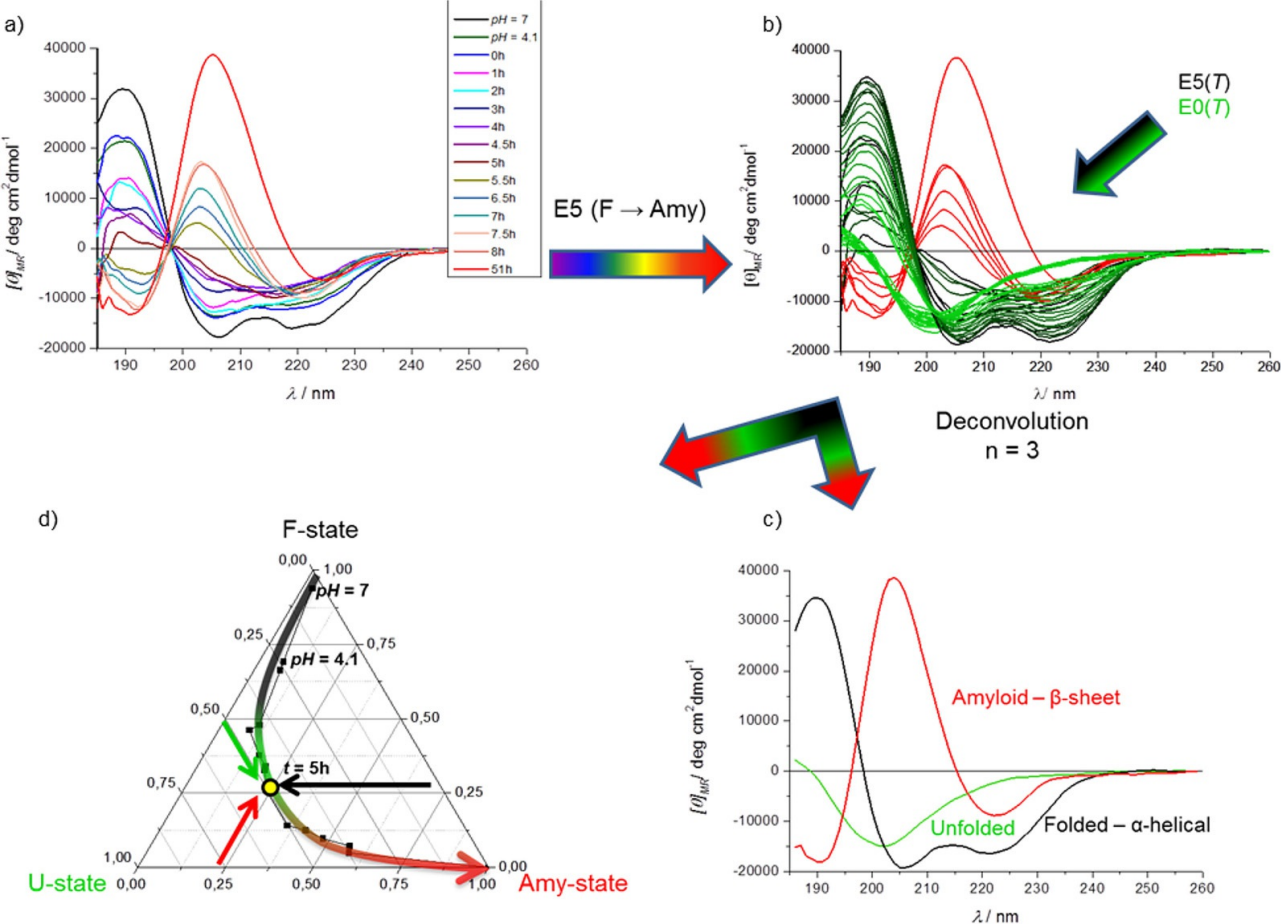
a) The C‐type FUV‐ECD of E5 (F‐state) gradually shifts as a function of time, as amyloid formation progresses (*c*_E5_=250 μm, *c*_NaCl_=50 mm, pH 4.1, stirring at *T*=37 °C). The final B‐type spectrum stands for the amyloid state (*t*=51 h) composed of β‐strands. b) An ensemble of the FUV‐ECD spectra of E5 forming amyloid (Panel A) was formed with those of the *T‐*dependent E0^[37]^and E5 (5<*T*(°C)<85) and deconvoluted into three pure component spectra c) standing for the F (black), U (green), and amyloid (red) states. d) The CCA+ resulted in the relative coefficients of these three pure ECD spectra and depicts the amyloid formation path in a Barycentric coordinate system: for example after 5 h the ratio is 26 % F, 50 % U, and 24 % Amy state (yellow dot) (c.f. Figure S7).

The overall path from the F‐ to the amyloid state is reported using a barycentric coordinate system (Figure 5) in which the gradual maturation of the amyloid as a function of time is visualized. At any point along the route, the ratio of the folded, unfolded, and amyloid states can be calculated from the deconvoluted spectral properties. As an example, the “mixed state” of [*p*_F_(*t*=5 h)=0.26, *p*_U_(*t*=5 h)=0.50, *p*_Amy_(*t*=5 h)=0.24] or simply (0.26, 0.5, 0.24) is shown on Figure 5 (see the “yellow dot” in Figure 5 d).

By using this mapping technique, three phases of the amyloid formation of E5 could be differentiated as i) initially the path runs parallel to the folded→unfolded axis, with no or marginal contribution of the amyloid state, corresponding to a misfolding phase, with the gradual accumulation of the unfolded/misfolded forms. ii) During the second phase the route runs parallel to the folded→amyloid axis, corresponding to the nucleation phase. During this phase a critical concentration of misfolded structures is reached, while the misfolded content remains nearly constant. F‐state diminishes while Amy‐states start to accumulate, iii) The third phase runs parallel to the unfolded→amyloid axis, where no further reversible unfolding takes place: the misfolded/unfolded structures are trapped by the growing amyloids, which is the elongation phase of the E5 amyloidosis.

### Fine‐tuning *c*_protein_ and *c*_salt_ affecting the amyloid formation

Although E5 can form amyloid at low salt (*c*_NaCl_<1 mm) and high protein concentrations (*c*_E5_>5–15 mm),^34^ under these extreme conditions amyloid formation is poorly reproducible. To locate physiologically more relevant and controllable amyloidogenic conditions on the Δ*G*=*f*(*T*,pH,*c*_protein_,*c*_ion_,*t*,) surface, both *c*_E5_ and *c*_NaCl_ variables had to be optimized at a sensible concentration range: 250<*c*_E5_<800 μm and 12.5<*c*_NaCl_<50 mm with *T* & pH set as: *T*=37 °C & pH 4.1. Fine‐tuning was completed with the HANABI system in a concerted manner leading to the identification of several concentration pairs for which amyloid formed and the transition was reproducible; namely, *c*_E5_ *=*250 μm and *c*_NaCl_ *=*50 mm, *c*_E5_ *=*500 μm and *c*_NaCl_=25 mm, *c*_E5_=800 μm and *c*_NaCl_=12.5 mm (with the solution constantly stirred) (Figure S8).

**Figure 6:**
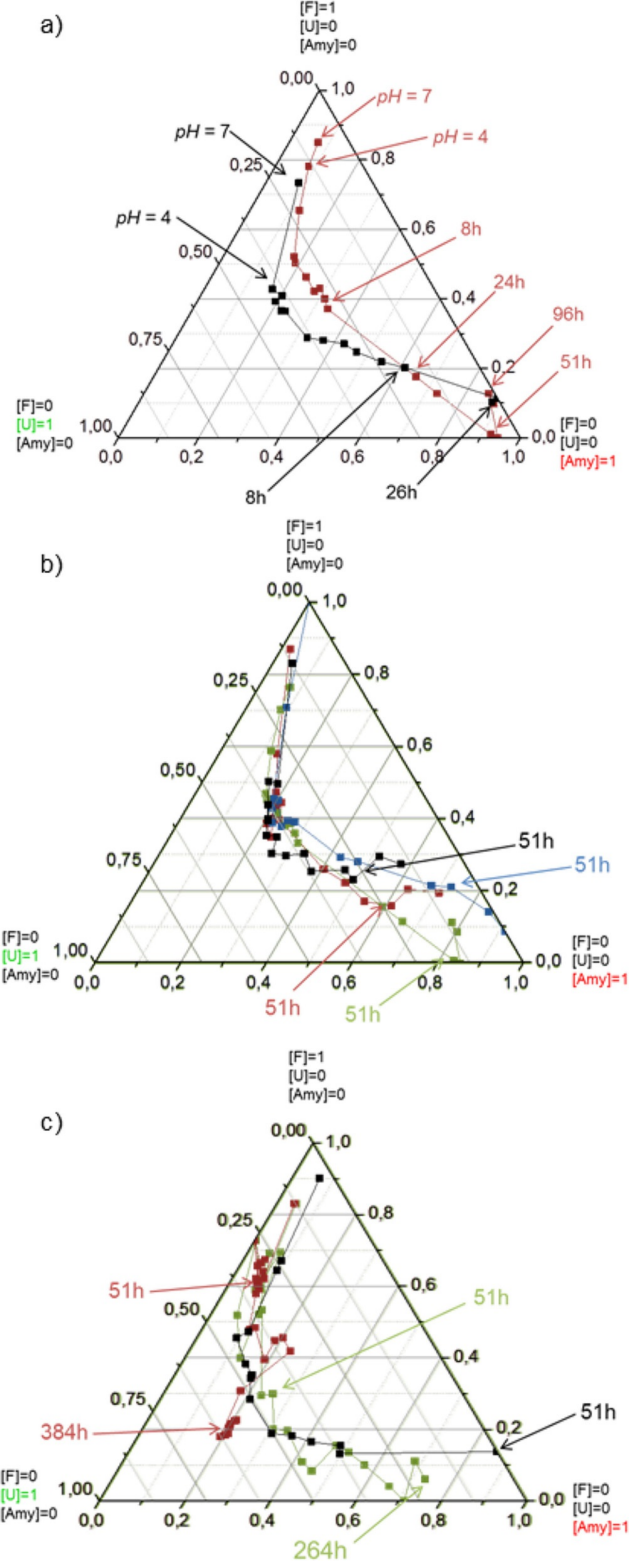
Amyloid formation completeness monitored by far‐UV ECD as a function of time with a) different protein (black: 800, claret: 400 μm) but same ion concentration (12.5 mm); b) same protein (400 μm) and different ion concentration (black: 50, claret: 25, green: 12.5, blue: 0 mm); c) same protein (250 μm) and the same ion concentration (50 mm) at different temperatures: *T*=23 (claret), 37 (black), and 47 °C (green).

As protein concentration increases, amyloid formation becomes more complete: [Amy]${}^{c{}_{E5}=400\;\mu m}$ (*t*=8 h)=0.34, [Amy]${}^{c{}_{E5}=800\;\mu m}$ (*t=*8 h)=0.61 even when salt concentration is low and fixed at *c*_NaCl_=12.5 mm (a condition more suitable for NMR) (Figure 6 a, Figure S9).

The rate of amyloid formation was also probed at a fixed protein concentration (e.g., *c*_E5_=400 μm) with increasing salt concentration (*c*_NaCl_: 0 mm, 12.5 mm, 25 mm, 50 mm) (Figure 6 b, Figure S10). We found that both misfolding and nucleation occurs at a similar rate. However, during the elongation phase clear differences were detected as a function of *c*_NaCl_. Upon stirring for three days (*t*=76 h) the amyloid ratio was found to be higher if salt concentration was lower: [Amy]${}^{c{}_{\mathrm{NaCl}}=0\;mm}$ =0.85, [Amy]${}^{c{}_{\mathrm{NaCl}}=12.5\;mm}$ =0.78, [Amy]${}^{c{}_{\mathrm{NaCl}}=25\;mm}$ =0.63, [Amy]${}^{c{}_{\mathrm{NaCl}}=50\;mm}$ =0.52. This finding, at first glance, could suggest that the easy way to avoid amyloid formation—at least for E5—is to use a high salt concentration. However, as we only detect amyloids of limited size by ECD (those that remain part of the solution), it is more likely that the larger salt concentration speeds up amyloid maturation and thus, eliminates shorter amyloid fragments from the solution—an assumption more in line with the literature data. Moreover, at the salt concentration used here (<100 mm), beside the nature of the anion and cation, specific ion‐binding to the polypeptide chain was also shown to contribute to the rate of amyloidosis.^46^, ^47^, ^48^ This could well be the case for E5 also: during MD simulations of the monomeric protein (both at pH 7 and pH 4.1 in 0.15 m NaCl solution) ca. 10 % of the all ion‐protein interactions involved the charged residues of the Glu^14^↔Arg^21^ salt bridge, which might contribute to the loosening of the hydrophobic core of E5 (Figure S11), influencing the ratio of the misfolded structures present.

### Temperature‐dependence revisited

The effect of temperature on the kinetics of amyloid formation was revisited by using the fine‐tuned conditions of amyloid formation (*c*_E5_=250 μm, *c*_NaCl_=50 mm and pH 4.1) (Figure 6 c, Figure S12). We found that the process is slower and incomplete both at 23 °C and 47 °C, compared to that of the physiological temperature (37 °C). It seems that at a high temperature the increased thermal motion disfavors self‐association: [Amy]^47 °C,51 h^=0.34≪[Amy]^37 °C,51 h^=0.86. At temperatures too low, the Brownian motion is significantly slower and thus fewer collisions occur and the misfolding propensity of E5 is lowered (see NMR data above): [Amy]^23 °C,51 h^=0.12.

### Verifying amyloidosis: NUV‐ECD measurements

**Figure 7:**
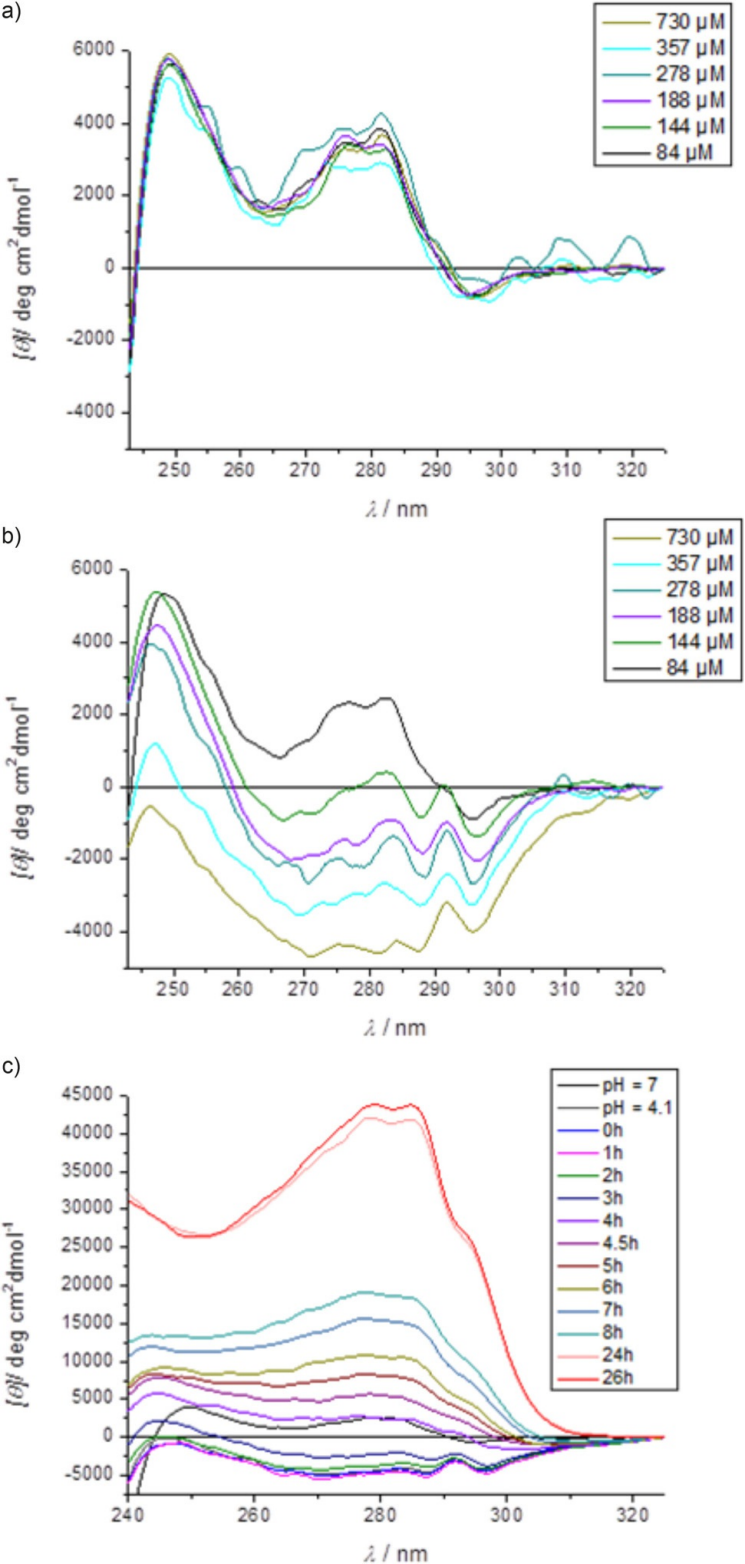
NUV‐ECD (240–325 nm) spectra reveal how conformers and interaction modes of Y^8^↔W^11^ aromatic residues change as function of the pH and time. At pH 4.1 b) the π–π interaction profile changes as protein concentration increases (84<*c*_E5_<730 μm at 25 °C, *c*_NaCl_≈0 mm), whereas it remains unchanged a) at pH 6.0. c) NUV‐ECD spectral shift signals indicating the changing interaction mode of Y^8^ & W^11^ as function of the time (0<*t*<26 h) during amyloid formation (*c*_E5_=800 μm, *c*_NaCl_=12.5 mm, pH 4.1, stirring at *T=*37 °C).

Amyloid transformation was monitored from the viewpoint of the Y^8^↔W^11^ aromatic interaction by acquiring NUV‐ECD data. At pH 6 the characteristic positive band at 283 nm (over the entire tested protein concentration range) is indicative of a shifted face‐to‐face π–π interaction between the two aromatic rings of the hydrophobic core of E5 (Figure 7 a). However, at pH 4.1 negative bands were detected in this spectral region (270<*λ*<290 nm) (Figure 7 b) and assigned to an edge‐to‐face π‐π interaction, signaling that misfolding of E5 concerns the relative re‐orientation of the two aromatic rings of the core. However, as amyloid transformation proceeds, gradually these negative bands are reverted and positive bands similar to those measured for the folded state at a slightly shifted position appear. As amyloid formation progresses, these bands intensify: after 26 h, band intensities are about 10 times higher than those of the initial F‐state (*Θ*_≈283 nm_: 4000→40 000) (Figure 7 a and c). This side chain restructuring coincides with the backbone changing from an α‐ to β‐state. Thus, we propose that the enhanced positive NUV‐ECD bands between 270<*λ*<290 nm no longer arise from a pairwise, intramolecular shifted face‐to‐face π–π interaction, but rather from the interstrand interaction of Tyr and Trp side chains packed tighter in the supramolecular assembly of the amyloid phase.

### The nature of the misfolded states

Amyloid formation proceeds at physiological temperature, but it can be quenched if cooled to 4 °C. Thus, a series of heteronuclear correlation spectra (^1^H‐^15^N‐HSQC) were recorded at 4 °C using samples retrieved at regular time intervals (every 30 min) during amyloid formation (Figure S13).

The ^1^H,^15^N‐chemical shifts of all residues were calculated by using Equation (1):

(1) $$\Delta \left(t\right)=\surd [\left(\delta H\left(t\right)\right){}^{2}+\left(\delta N\left(t\right)/6,51\right){}^{2}]$$

as function of the time (0<*t*<24 h). Residues of larger changes than the average chemical shift (*t*_end_−*t_0_*>0.047 ppm) are those of the α‐helix (E^2^, E^3^, V^5^, L^7^, I^9^, Q^10^, K^13^), signaling that amyloid formation affects this region. Residues of the helix were found to be the most temperature sensitive as well (Figure S2), indicating that this region is assailable as soon as the shielding efficacy of the Trp‐cage fold against water gets reduced. This region (segment ‐R^6^LYIQWL^12^‐) was also predicted to be the most amyloidogenic of the sequence by CamSol.^49^ Therefore, we propose that the α‐helix itself is the seed of amylogenic nucleation. As its misfolding and transient unwinding allows the reorientation of the aromatic side chains, di‐ and oligomers of the misfolded monomers can interact via their exposed hydrophobic cores.

**Figure 8:**
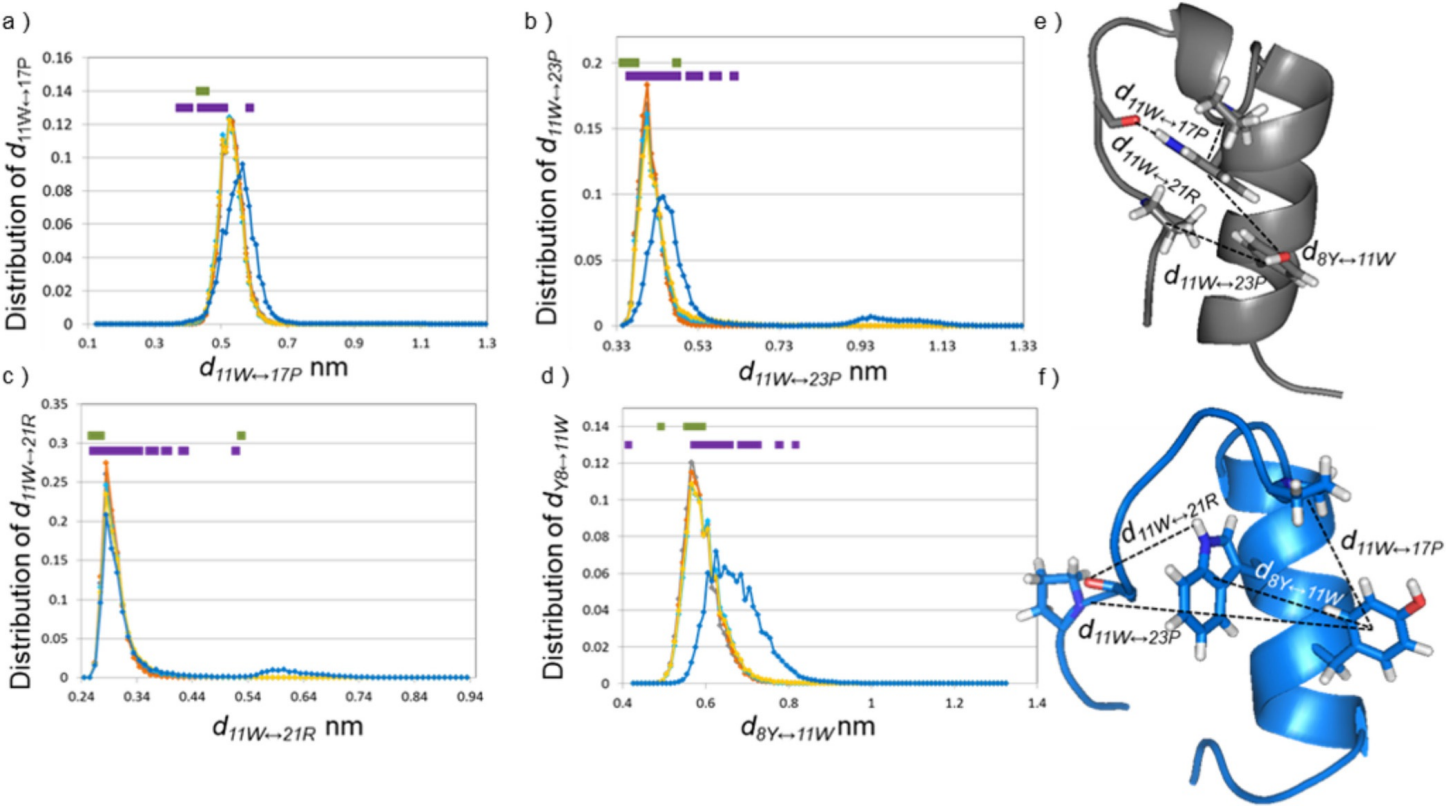
The distributions of conformational measures introduced to signal the degree of misfolding of E5. a–d) The distribution of the selected measures for the MD trajectories (gray: pH 7, E5[^1^E_−1_,^2^E_−1_,^3^E_−1_,^14^E_−1_]; orange: pH 4.1 E5[^1^E_0_,^2^E_0_,^3^E_0_,^14^E_−1_]; light blue: pH 4.1 E5[^1^E_0_,^2^E_0_,^3^E_−1_,^14^E_0_]; yellow: pH 4.1 E5[^1^E_0_,^2^E_−1_,^3^E_0_,^14^E_0_]; blue: pH 4.1 E5[^1^E_−1_,^2^E_0_,^3^E_0_,^14^E_0_] are plotted, whereas the distribution of the selected measures for NMR structures (green: pH 7, purple: pH 4.1) are indicated for every structure above the MD distribution curves in a linear 1D manner. The measure of the E5[^1^E_−1_,^2^E_0_,^3^E_0_,^14^E_0_] microstate is slightly different from the other microstates; moreover, a minor population of the E5[^1^E_−1_,^2^E_0_,^3^E_0_,^14^E_0_] microstate shows considerably distinct properties. Table 1 contains a limit for each measure that can be used to distinguish/separate the divergent/misfolded population of the E5[^1^E_−1_,^2^E_0_,^3^E_0_,^14^E_0_] microstate. e) NMR calculated folded structure (gray), and f) one of the MD‐based misfolded (blue) structures are shown to visualize the conformational differences. Additional distributions of the selected measures are shown in Figure S15.

Amyloid formation is optimal at pH 4.1, where the overall charge of E5 is near +2 (Figures 1, 2, and 3) and thus, on average three out of the four Glu side chains are protonated. To identify the most likely protonation state to trigger misfolding, MD simulations were carried out. At pH 7 only one possible protonation motif, that of E5[^1^E_−1_,^2^E_−1_,^3^E_−1_,^14^E_−1_] exists, while at pH 4.1 as many as four (E5[^1^E_0_,^2^E_0_,^3^E_0_,^14^E_−1_]; E5[^1^E_0_,^2^E_0_,^3^E_−1_,^14^E_0_]; E5[^1^E_0_,^2^E_−1_,^3^E_0_,^14^E_0_]; E5[^1^E_−1_,^2^E_0_,^3^E_0_,^14^E_0_]) different protonation patterns have to be considered, which we will refer to as microstates. Conformational descriptors were selected to measure the extent of misfolding (see Methods, Table 1 and Figure 8, Figure S14).

**Table 1:** Conformational measures introduced to signal the degree of misfolding of E5. Each measure type a critical value or critical interval was specified to characterize the divergent/misfolded population of the ominous E5[^1^E_−1_,^2^E_0_,^3^E_0_,^14^E_0_] microstate.

	Measure type	Description of the measure	Critical value of the measure
i	*d*_11W↔17P_	distance between Trp^11^ & Pro^17^	*d*_11W↔17P_>5.7 Å
ii	*d*_11W↔23P_	distance between Trp^11^ & Pro^23^	*d*_11W↔23P_>4.7 Å
iii	*d*_11W↔21R_	distance between NH of Trp^11^ and CO of Arg^21^	*d*_11W↔21R_>6.0 Å
iv	*d*_14E↔21R_	distance between donor of Arg^21^ and acceptor of Glu^14^	
v	*μ*	torsion angle between the main axis of the core α‐helix and of the polyPro of the C terminus	*μ*>45°
vi	*d*_8Y↔11W_	distance between Tyr^8^ & Trp^11^	*d*_8Y↔11W_>7.0 Å
vii	*α*	angle between the planes of the aromatic rings	*α*>70°
viii	*θ*	elevation of the aromatic rings	*θ*>6.5°
			40>*θ*>50°
			80>*θ*>90°
ix	*φ*	azimuthal angle of the aromatic rings	*φ*>320°

**Figure 9:**
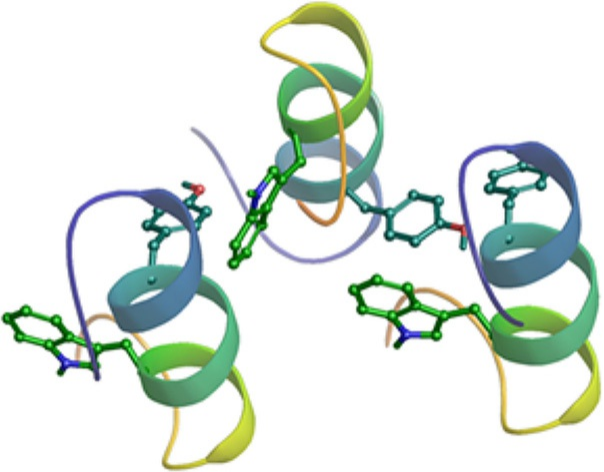
Possible self‐aligning mode of a conformer of the MD simulation. The antiparallel orientation of the helices is due to Coulombic interactions operative at pH 4.1 in the selected microstate. Aromatic–aromatic (Y–W) interaction driven backbone pairing of the misfolded molecules.

The simulations of four microstates provided equilibrium ensembles quite similar to that of the folded structure derived from NMR data: those of E5 _pH 4.1_[^1^E_0_,^2^E_0_,^3^E_0_,^14^E_−1_], E5 _pH 4.1_[^1^E_0_,^2^E_0_,^3^E_−1_,^14^E_0_] and E5 _pH 4.1_[^1^E_0_,^2^E_−1_,^3^E_0_,^14^E_0_]. In contrast, a single microstate, that of E5 _pH 4.1_[^1^E_−1_,^2^E_0_,^3^E_0_,^14^E_0_] was significantly different from any of the above when descriptors *i*−*ix* were evaluated, this state shows strong resemblance to the loosened NMR structural ensemble measured at pH 4.1. We found that in case of E5 _pH 4.1_[^1^E_−1_,^2^E_0_,^3^E_0_,^14^E_0_] both *d*_11W↔17P_, and *d*_11W↔23P_, distances are lengthened by 0.4 and 0.3 Å, respectively, and in some conformers the *d*_11W↔21R_ distance shifted from 2.85 to 5.85 Å, indicating the appearance of less compact protein folds. Furthermore, as the distribution of *μ* gets wider, the ‐PPP^24^‐ segment twists more often with respect to the main axes of the α‐helix. In parallel, *d*_*8*Y↔11W_ increases by 0.9 Å. The relative orientation of the aromatic side chains changes as well: measured *θ* shows a more diverse distribution relative to the other microstates, while the values of *α* and *φ* increase slightly. Summing up, we might say that E5 _pH 4.1_[^1^E_0_,^2^E_0_,^3^E_0_,^14^E_−1_] protonation microstate has an enhanced backbone conformational freedom and a significantly rearranged hydrophobic core, with respect to all the others. The Trp‐cage gets occasionally unfolded, giving rise to unshielded aromatics and exposed backbone amide groups, ready for self‐association and subsequent amyloid formation (Figure S14), though these events are transient, and misfolding is temporary. However, it should be noted that MD simulations carried out here consider isolated molecules. The exposed aromatic and hydrophobic side chains create a “sticky” interaction center for E5, quite as the free β‐edges that appear transiently on the surface of locally misfolded large, globular proteins and become initiators of aggregation. Thus, when collisions among similarly loosened conformers of E5 are also considered, the described changes might become sufficient to create the first di‐ and oligomer nuclei of aggregates (Figure 9). These findings also explain why stirring is necessary for amyloid formation of E5; given that only one of the four possible protonation patterns of pH 4.1 produces a “misfolded enough” conformer, a great number of collisions are required for successful nucleation.

### Building a model of the amyloid state

To obtain a picture of the amyloid state, MD simulations were carried out for models of four‐β‐strand clusters (E5)_4_ using three different protonation motifs: (E5 _pH 2_[^1^E_0_,^2^E_0_,^3^E_0_,^14^E_0_, C‐term_0_])_4_, (E5 _pH 4.1_[^1^E_−1_,^2^E_0_,^3^E_0_,^14^E_0_])_4_ and (E5 _pH 7_[^1^E_−1_,^2^E_−1_,^3^E_−1_,^14^E_−1_])_4_ (four‐stranded models were shown to be sufficiently large for modeling aggregation nuclei in various different systems^50^, ^51^, ^52^). We have probed three different relative offsets, namely that of “YI”, “YK”, and “YW” (Figure S16 A, B, and C) for each.

From the three probed offsets, YW turned out to be both the most stable at pH 4.1 and the most sensitive to pH shift (since both at pH 2 and 7, the folded conformation is most stable and practically no amyloid formation could be detected, we expected the most realistic model to be significantly more stable at pH 4.1) (Figure S17). Considering that in the ‐PPP^24^‐ segment *φ* ^Pro^ must be ca. −60° and thus this part of the sequence is unlikely to form an extended β‐sheet structure (with *φ*≈−145, *ψ*≈+145°), in case of the YW offset 18 pairs of interstrand H‐bonds could be formed between residue 1–18 in total. In YW_pH 4.1_ on average 10 out of 18 H‐bonds are present during the MD simulation between the two middle chains, typically involving residue‐pairs such as V^5^‐E^14^, L^7^‐L^12^, I^9^‐Q^10^, W^11^‐Y^8^, and K^13^‐R^6^. These interstrand H‐bonds confirm that an aggregation core can indeed form between residues 5–14, as suggested above. Furthermore, in this arrangement of YW_pH 4.1_ an aromatic ladder forms between the Y^8^ and W^11^ side chains of adjacent β‐strands, as they come in close proximity (*d*_8Y↔11W_ <6 Å as measured between the center of the rings) (Figure S16 D). The aromatic ladder enables the formation of the shifted face‐to‐face π–π interaction expected based on ECD spectroscopic data. Interestingly, the derived orientation and spacing of the Tyr and Trp side chains here is also quite reminiscent of the aromatic clusters created by mutations within the central single‐layer β‐sheet of *Borrelia* outer surface protein A (OspA) to probe the structure‐ordering capacity of such residues.^53^

**Figure 10:**
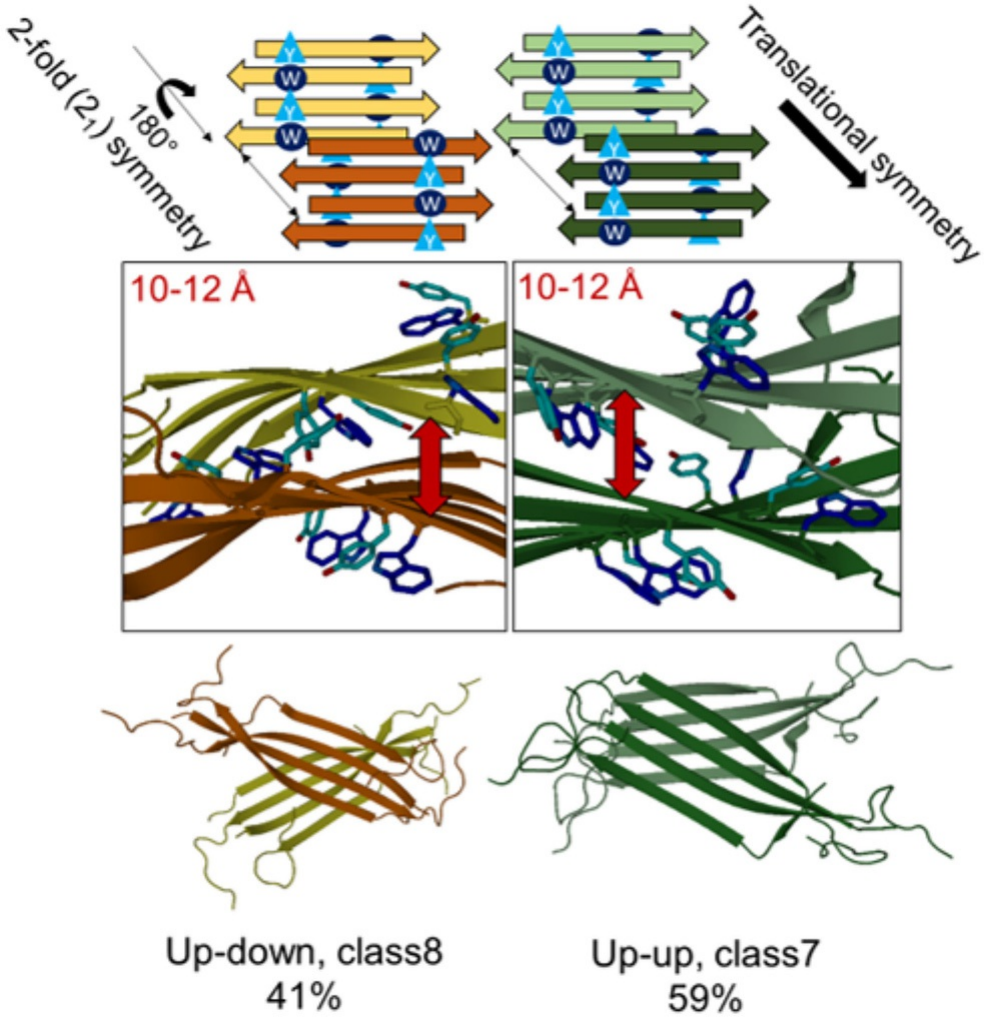
The two basic topologies found by docking (E5)_4_ amyloid seeds are the antiparallel equifacial antisymmetric up–down (class 8) with a twofold symmetry and equifacial antisymmetric up–up (class 7) with translational symmetry. The schematic illustrations of the basic amyloid layout shown have the aromatic residues highlighted: above, Tyr: light‐blue triangle and Trp: dark‐blue circle. Twisted amyloid β‐sheets (below) and 90°‐rotated plus enlarged view of the aromatic residues are shown in the middle: Tyr (light‐blue) and Trp (dark‐blue) with oxygen atoms red and nitrogen (middle blue). Red double arrows show the specific distance of the associated β‐sheets.

Using the mid‐structures of six different clusters (see Methods) of the MD simulation of (E5 _pH 4.1_)_4_ YW as possible amyloid seeds, we also probed different inter‐sheet arrangements by using a protein‐protein docking algorithm. The near‐parallel arrangements thus obtained could be grouped into two fundamentally different topologies (using the nomenclature introduced by Eisenberg and co‐workers^54^): those of class 7 (equifacial, antisymmetric, up‐up) with translational symmetry, and class 8 (antiparallel, equifacial, antisymmetric, up‐down) with twofold symmetry (Figure 10).

In the derived β‐sheet‐dimers, ((E5 _pH 4.1_)_4_)_2_, the average distance between the β‐sheets separated by the zipper interface varies between 10–12 Å, in line with the 8–10 Å experimental values determined for various amyloid structures.^55^, ^56^, ^57^, ^58^, ^59^ Furthermore, in this sandwich form, the intersheet aromatic side chains may approach each other even closer than the intrasheet 6 Å described above (Figure S18). We found that (E5 _pH 4.1_)_4_ seeds with a slightly twisted backbone find partners more readily to form the sandwich structure than those with fully extended backbones, in accordance with the general view that twisted filaments are more stable and thus more prevalent in nature.^60^ These findings further support our model for the aggregation of E5 as nucleated by the antiparallel oriented β‐sheets formed between the most aggregation‐prone segment of the sequence, the ‐V^5^RLYIQWLK^13^‐ unit.

## Conclusions

Concerning E5, the Exenatide variant studied here, we found that its amyloid aggregation is most likely triggered by the transiently exposed aromatic and hydrophobic side chains of loosened—misfolded—conformers, which create a center for intermolecular associations. The clusters thus created provide stabilization (spatial closeness for sufficient time) for the much slower process of α‐helix to coil and then to extended β‐strand transition, which seems to be highly unfavorable for the monomeric forms but eventually leads to the formation of a β‐structured amyloid nucleus. The amyloidosis of E5 thus follows the nucleated growth mechanism.^42^, ^43^

Studying the early phases of amyloid formation is crucially important both for understanding the initialization of various pathophysiological processes and for aiding the design of non‐toxic peptide medications that will not become initiators of such processes themselves. We derived a new monitoring technique of amyloid progression that can be applied even if the amyloid in question is ThT silent—using simple ECD measurements. Decomposition of the spectra and its barycentric representation on the folded‐unfolded‐amyloid potential energy surface of the amyloidic transition can be applied to filter out potentially harmful sequences from development. Generally, it can be concluded that the lowest possible salt concentration, low temperatures, and the absence of agitation can prolong the shelf‐life of any polypeptide and protein medications, but, somewhat disturbingly, we also found that the optimum temperature for E5 amyloidosis coincides with our body temperature and requires well below physiological salt concentration. This just underlines how important it is to test the aggregation propensity of any drug candidates.

## Experimental Section

**Protein expression and purification**: The E5 miniprotein was produced by bacterial expression using the previously published protocol.^33^

**Preparation of amyloid form of E5 miniprotein**: The lyophilized E5 samples were dissolved in distilled water. First, the pH of protein solution was adjusted to pH 7 with 0.1 m NaOH solution (Orion Star A211 pH meter (Thermo Scientific™)) then decreased to 4.1 or lower (pH‐dependent amyloid formation) with 0.1 m HCl solution. Finally, desired NaCl concentration was adjusted with concentrated NaCl solution. The protein solution was stirred (with magnetic stirrer) and incubated at target temperatures. At a given time, 10‐fold diluted sample was measured with ECD spectroscopy. The concentration of the diluted sample was determined with a NanoDrop Lite Spectrophotometer (Thermo Scientific™) at 280 nm.

**NMR experiments**: Datasets were collected with a 16.4 T Bruker Avance III spectrometer equipped with a 5 mm inverse TCI probe‐head with *z‐*gradient. Spinlock (d9) for ^1^H‐^1^H TOCSY was 80 ms, while the mixing time (d8) of 150 ms was taken for ^1^H‐^1^H NOESY spectra. NMR structure calculations were performed and refined by cooperative use of CcpNmr Analysis 2.4.1., Aria 2.0^61^ and CNS Solve 1.2.^62^

**Molecular dynamics simulations**: MD simulation were carried out as implemented in GROMACS59, using the AMBER‐ff99SBildnp* force field. The systems were solvated with TIP3P water molecules in dodecahedral boxes with a size allowing 10 Å between any protein atom and the box. Trajectories of 600 ns ((E5)_4_ models) to 1000 ns (E5 monomers) NPT simulations with a 2 fs time step at 310 K and 328 K and 1 bar were collected (with snapshots at every 4 ps).

**Electronic circular dichroism spectroscopy**: Far‐ and near‐UV ECD measurements were carried out with a Jasco J810 spectrophotometer. The temperature at the cuvette was controlled with a Peltier‐type heating system.

## Conflict of interest

The authors declare no conflict of interest.

## Supporting information

As a service to our authors and readers, this journal provides supporting information supplied by the authors. Such materials are peer reviewed and may be re‐organized for online delivery, but are not copy‐edited or typeset. Technical support issues arising from supporting information (other than missing files) should be addressed to the authors.

Supplementary
