# Supplementary material for: The Route from the Folded to the Amyloid State: Exploring the Potential Energy Surface of a Drug‐Like Miniprotein
Source: Chemistry. 2019 Dec 27;26(9):1968–78. doi: 10.1002/chem.201903826 (PMC7028080; doi:10.1002/chem.201903826)
Supplement: Supplementary file 1 — Supplementary [file CHEM-26-1968-s001.pdf]

CHEMISTRY
A European Journal

Supporting Information

The Route from the Folded to the Amyloid State: Exploring the Potential Energy Surface of a Drug-Like Miniprotein

Nóra Taricska,[a] Dániel Horváth,[a] Dóra K. Menyhárd,[a] Hanna Ákontz-Kiss,[a] Masahiro Noji,[b] Masatomo So,[b] Yuji Goto,[b] Toshimichi Fujiwara,[b] and András Perczel*[a]

chem_201903826_sm_miscellaneous_information.pdf

Supporting Information

Methods

NMR experiments: Datasets were collected on a 16.4 T Bruker Avance III spectrometer equipped with a 5 mm inverse TCI probe-head with z-gradient. Each sample contained 700-900 μM of E5 miniprotein, 10% D_2O, 0.1 mM NaN_3 and DSS as an internal proton reference standard. The concentrations were determined by Nanodrop. The sample pH was set between *pH*= 7.1 and 2.0 by using 0.1 M NaCl or HCl and the measurements were recorded from 277.0 K to 321 K by equal steps of 11 K. Spinlock (d9) for ^{1}H-^{1}H TOCSY was 80 ms, while the mixing time (d8) of 150 ms was taken for ^{1}H-^{1}H NOESY spectra. Raw spectra were processed by TopSpin 3.2 and the homonuclear proton NMR assignations were made using CcpNmr Analysis 2.4.1[1]. The NMR structure calculations were performed and refined by cooperative use of CcpNmr Analysis 2.4.1., Aria 2.0[2] and CNS Solve 1.2.[3] The NOE cross-peak intensities were converted into distance. The peak volume scaling factor is defined such that the peak list's average volume corresponds to the default 3.2 Angstroms. Each ensemble was refined in water and contains 50 structures.

Molecular dynamics simulations: MD simulation were carried out as implemented in GROMACS59, using the AMBER-ff99SBildnp* force field. The systems were solvated with TIP3P water molecules in dodecahedral boxes with a size allowing 10 Å between any protein atom and the box. The total charge was neutralized and physiological salt concentration was set using Na^+ and Cl^- ions. Energy minimization of starting structures was followed by sequential relaxation of constraints on protein atoms in three steps and an additional NVT step (100 ps) to stabilize pressure. Trajectories of 600 ($(E5)_4$ models) - 1000 ns (E5 monomers) NPT simulations with a 2 fs time step at 310 K and 328 K and 1 bar were collected (with snapshots at every 4ps). The last 500 ns of the trajectories were clustered fitting the main-chain atoms using a 1 Å cutoff in case of the monomers, while the last 400 ns of the trajectories of the tetramer sheets were clustered using a 1.7 Å cutoff fitting the main-chain atoms of the seed segment (residues 6-13). 112 clusters were found (95% of the snapshots are in 32 clusters). For docking 4-stranded seed models to one-another, we choose six different 4-stranded sheet conformer (middle structures of six different clusters from the MD simulation of the ($\boldsymbol{E5}_{pH=4.1}[^{1}E_{-1}, ^{2}E_{0}, ^{3}E_{0}, ^{14}E_{0}])_4$ microstate with an YW offset) which were docked to another identical 4-stranded sheet, using Piper[4]. During docking the side chains were rigid, 10000 ligand rotations were probed and the 30 largest clusters were chosen for further analysis. We selected manually the most relevant amyloid-like structures from the clusters (less than 45° angle between the strands, the plane of the sheets close to parallel): these were 10-24% of the total 30 clusters. The distance of the β-sheets was measured as the backbone distance the aromatic residues of the two middle chains between the two opposite sheets of the amyloid seed region (residues 6-13).

Electronic circular dichroism spectroscopy: Far- and near-UV ECD measurements were carried out on a Jasco J810 spectrophotometer in 1.0 and 10 mm quartz cuvettes. Typically we used spectral scanning speed of 50 nm/min with 1 nm bandwidth and the 0.2 nm step resolution over wavelength range 185-260 nm (far-UV) and 240-325 nm (near-UV) with three and four scans averaged for each spectrum. The temperature at the cuvette was controlled by Peltier-type heating system (temperature dependent measurements were made from 5 °C to 85 °C in 5 °C steps and the amyloid samples were measured at 25 °C). The raw ellipticity data were converted into mean residue molar ellipticity units ($[\vartheta]_{MR}$/ deg*cm^2*$dmol^{-1}$) for the far-UV region and molar ellipticity ($[\vartheta]$/ deg*cm^2*$dmol^{-1}$) for the near-UV region.

CCA+ deconvolution: During deconvolution of amyloid formation we used the temperature dependent ECD spectra of E5 (as folded) and E0 (as unfolded) miniprotein. (These spectra were measured from 5 °C to 85 °C for 5 °C step (17 spectra/ miniprotein)). This resulting matrix was deconvoluted into 3 components and then reported in a barycentric coordinate system.

TEM measurement: Sample solution (5 μL) was spotted onto a collodion-coated copper grid (Nisshin EM Co., Tokyo, Japan). After 1 min, the remaining solution was removed with filter paper and 5 μL of 2 % (w/v) ammonium molybdate was spotted onto the grids. After 1 min, the remaining solution was removed in the same manner. TEM images were obtained with a Hitachi H-7650 transmission electron microscope (Tokyo, Japan) with a voltage of 80 kV.

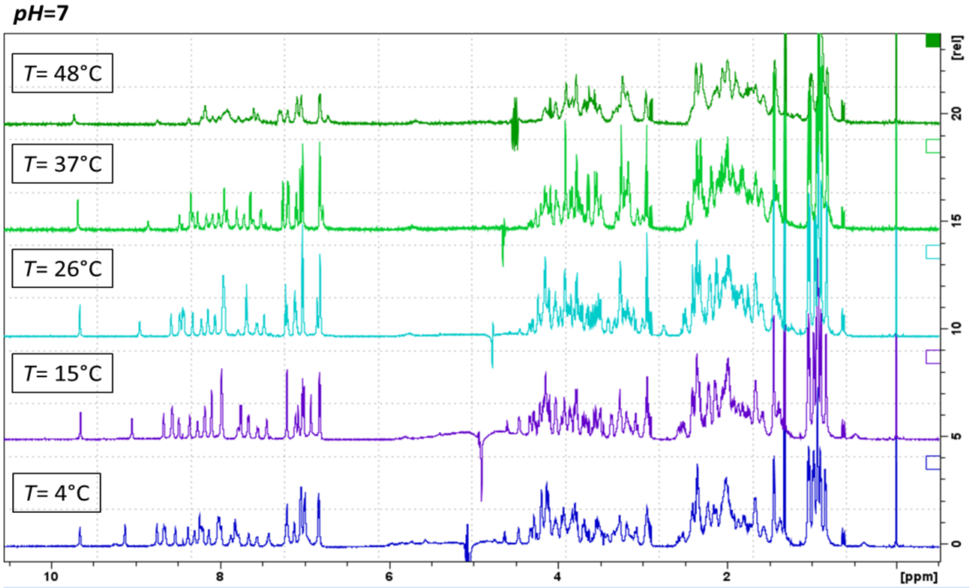

SFigure 1. ^{1}H-NMR spectra of E5 (*pH*= 7, 0.78 mM) as a function of the *T*(°C). The dispersed chemical shifts indicate a single time-average compact fold of the protein between 4 to 37 °C, signaling some reversible unfolding at 48 °C and above (data not shown).

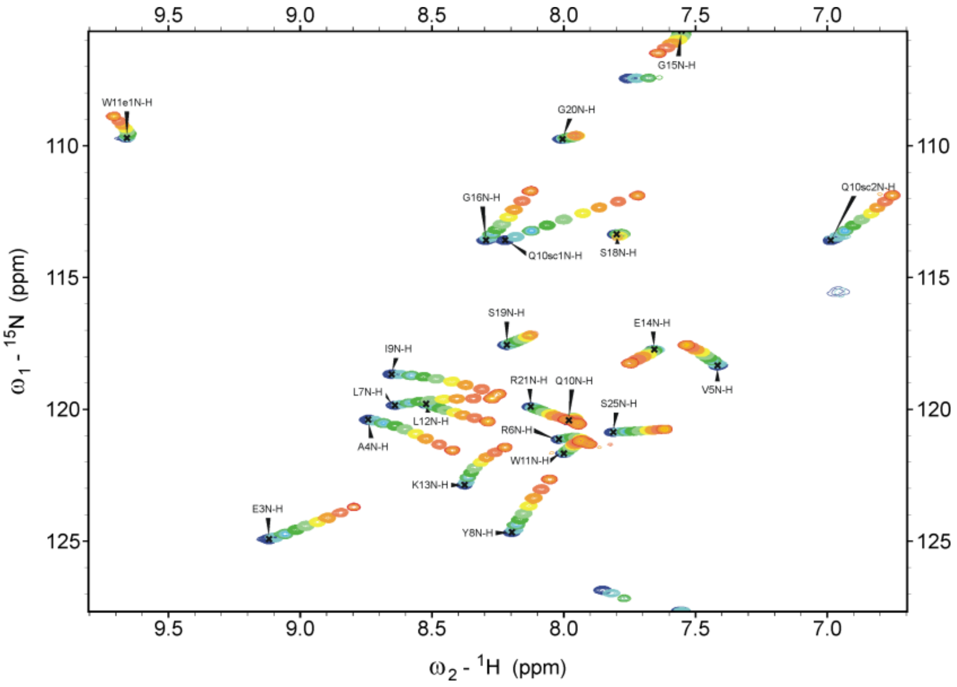

SFigure 2. The temperature (5 ≤ *T*(°C) ≤ 55) dependent ^{1}H-^{15}N-HSQC of E5 *pH*= 7. The curved (non-linear) temperature induced NH chemical shift changes are indicative of the I-state of selected amino acid residues: moderate for A^{4}, V^{5}, Y^{8} and pronounced **non-linearity** is measured for L^{7}, I^{9}, K^{13} residues. The F-sate structure of E5 colored according to the prevalence of an I-state, H-EEEAVRLYIQWLKEGGPSSGRPPPS-OH: lower probability orange, higher red.

Thermal unfolding ← → pH remodelling

Tyr8	48°C, pH=7	37°C, pH=7	15°C, pH=7	15°C, pH=5	15°C, pH=4	15°C, pH=2
Val5	2	3	3	-	1	6
Trp11	-	-	1	-	-	1
Leu12	4	7	6	3	3	6
Pro23	2	2	6	-	2	7
Pro24	5	4	7	1	1	9

Trp11	48°C, pH=7	37°C, pH=7	15°C, pH=7	15°C, pH=5	15°C, pH=4	15°C, pH=2
Tyr8	-	-	2	-	-	2
Gly16	-	-	4	-	-	7
Pro17	-	7	12	8	6	17
Arg21	5	8	11	3	3	10
Pro22	-	-	2	-	-	3
Pro23	1	4	18	4	4	10
Pro24	-	1	3	-	-	4

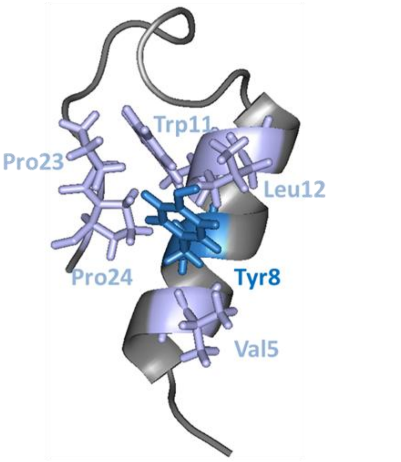

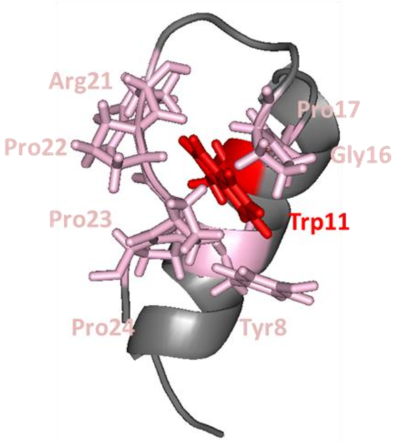

SFigure 3. The residue specific interaction matrix indicating the 3D-fold compactness of E5 as function of the *pH* and *T*: the total number of distance restrains i→(i+3) and longer) between selected residues of the protein core (Tyr^{8}↔Xxx and Trp^{11}↔Xxx) are depicted.

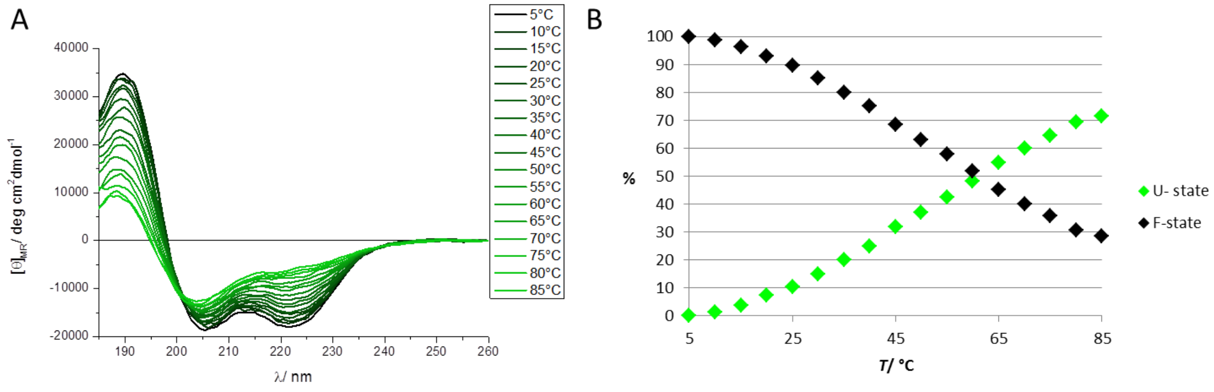

SFigure 4. A) Temperature dependent far-UV ECD spectra of E5 miniprotein at *pH*= 7.0. **B)** The ensemble deconvolution of the 17 spectra gives a quantitative measure of the folded and unfolded fraction of the protein at any temperature (5 ≤ *T*(C°) ≤ 85).

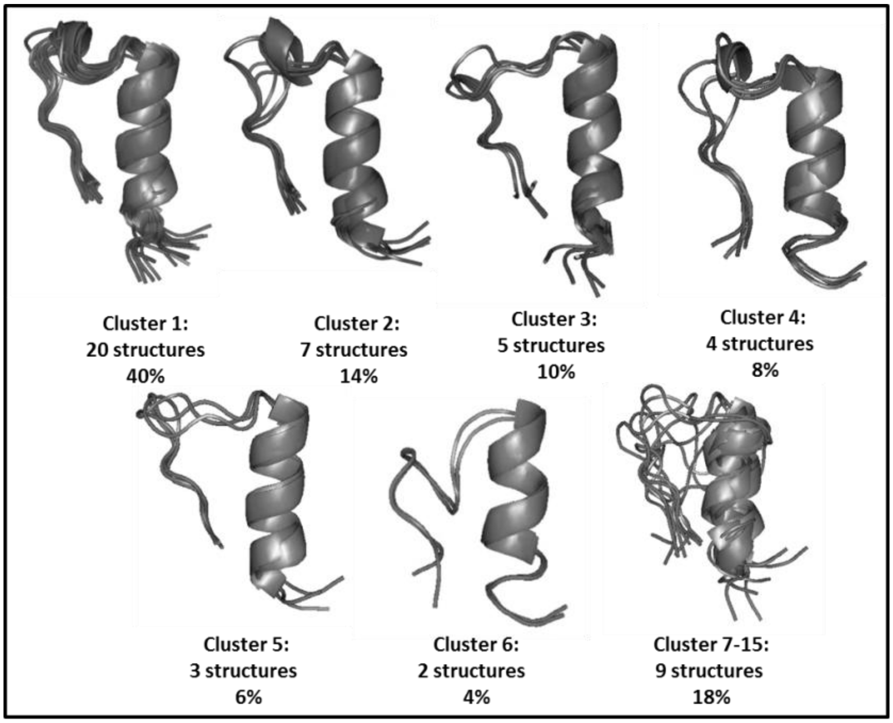

SFigure 5. The best 50 E5 structures determined by NMR at the amyloidogenic *pH*= 4.1 (*T*= 15 °C). 339 restrains in total ($\Sigma NOE^{i \rightarrow (i+5<)}$= 22), with an 1.5 nm cutoff for all backbone atoms RMSD were used for clustering.

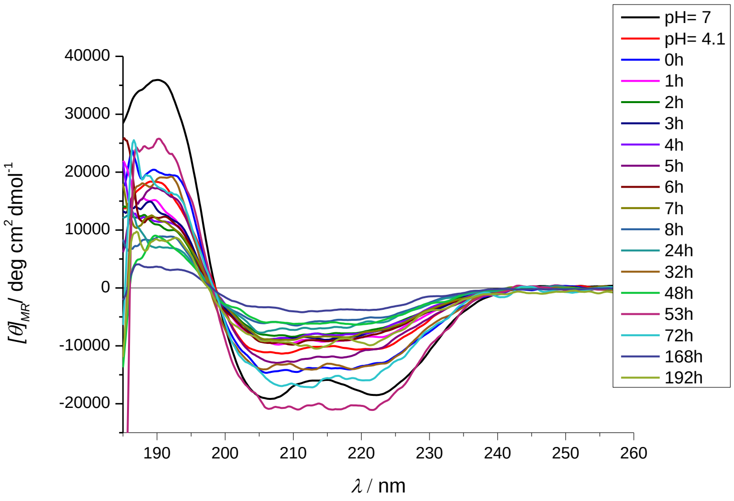

SFigure 6. In the absence of stirring or sonication the gradual decay of the far-UV ECD spectrum intensities of E5 was detected: c_{E5}= 250 µM, c_{NaCl} = 50 mM, *pH*= 4.1, *T*= 37 °C.

t/h	p_F	p_U	p_{Amy}
0	0.69	0.24	0.07
1	0.48	0.41	0.11
2	0.46	0.45	0.09
3	0.38	0.46	0.16
4	0.34	0.46	0.2
4.5	0.33	0.47	0.2
5	0.26	0.50	0.24
5.5	0.14	0.50	0.36
6.5	0.12	0.46	0.42
7	0.1	0.42	0.48
7.5	0.07	0.36	0.57
8	0.05	0.37	0.58
51	0	0	1.0

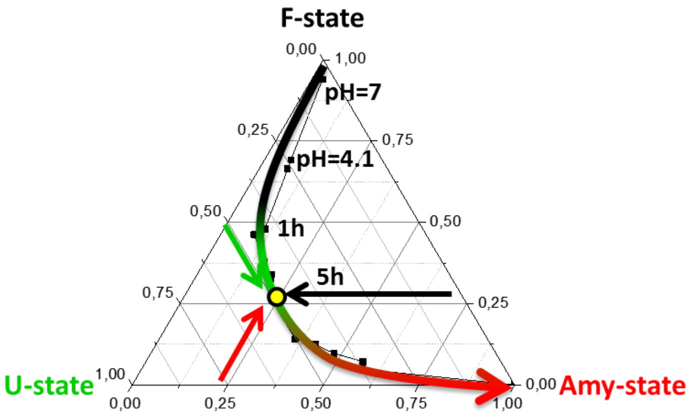

SFigure 7. A CCA+ resulted in relative coefficients of the pure ECD curves tabulated and reported in a Barycentric coordinate system (conditions used were: c_{E5}= 250 μM, c_{NaCl}= 50 mM, *pH*= 4.1, and stirring at *T*= 37 °C).

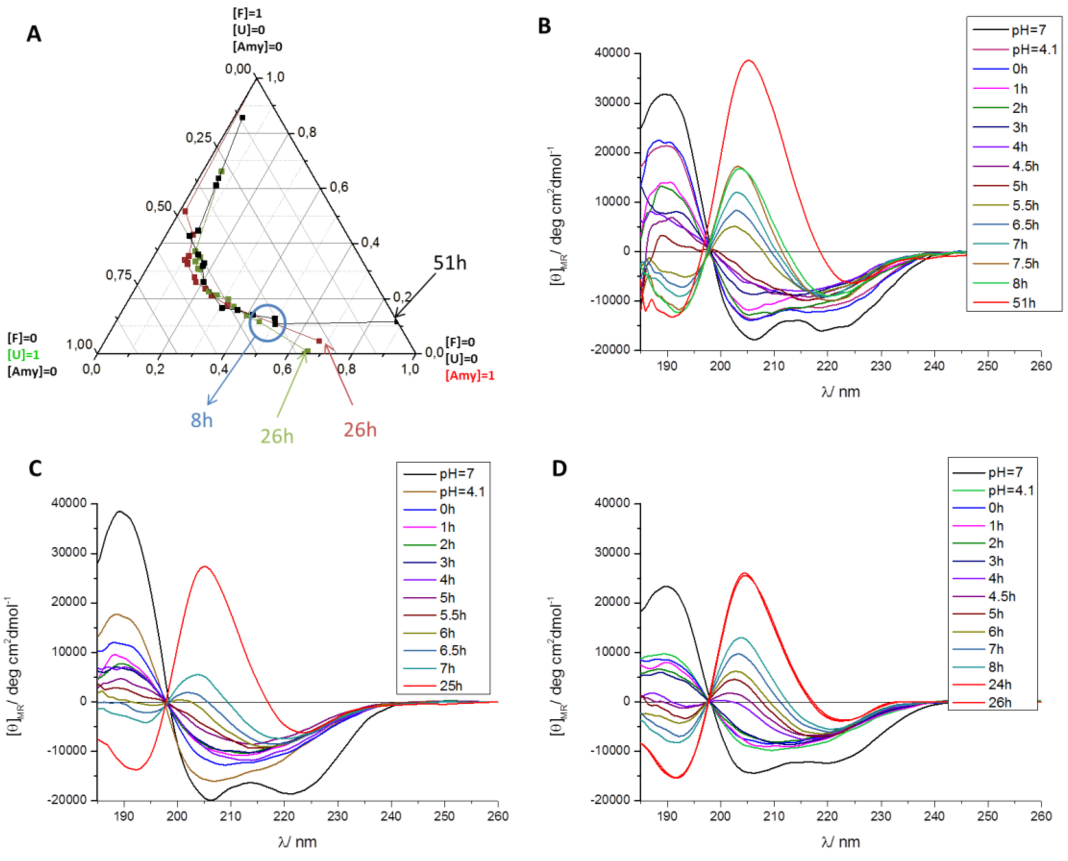

SFigure 8. *Amyloid* forms at a large concentration range: from c_{E5}= 250 *μM & c_{NaCl}= 50 mM* up to c_{E5}= 800 μM & c_{NaCl} = 12.5 mM. (**A**) The pinpointed pathways and formation rate looks similar *[c_{E5}= 250 μM & c_{NaCl}= 50 mM (black) and* c_{E5}= 500 μM & c_{NaCl}= 25 mM, *(claret) and* c_{E5}= 800 μM & c_{NaCl}= 12.5 mM, *(green) (pH*= 4.1 & *stirring at T= 37 °C)]*. Conformational changes determined by FUV-ECD as function of the time, during amyloid formation at c_{E5}= 250 μM & c_{NaCl}= 50 mM (**B**) and c_{E5}= 500 μM & c_{NaCl}= 25 mM, (**C**) and c_{E5}= 800 μM & c_{NaCl}= 12.5 mM (**D**) at *pH*= 4.1, stirring at *T*= 37 °C.

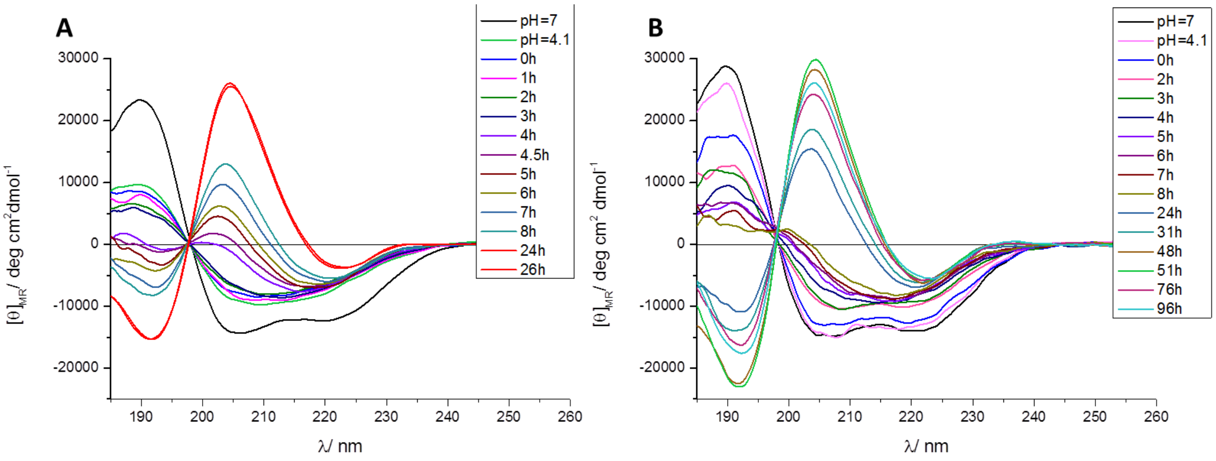

SFigure 9. Conformational changes determined by FUV-ECD as function of the time, during amyloid formation at c_{E5}= 800 µM, c_{NaCl}= 12.5 mM (**A**) and c_{E5}= 400 µM, c_{NaCl}= 12.5 mM (**B**) at *pH*= 4.1, stirring at *T*= 37 °C.

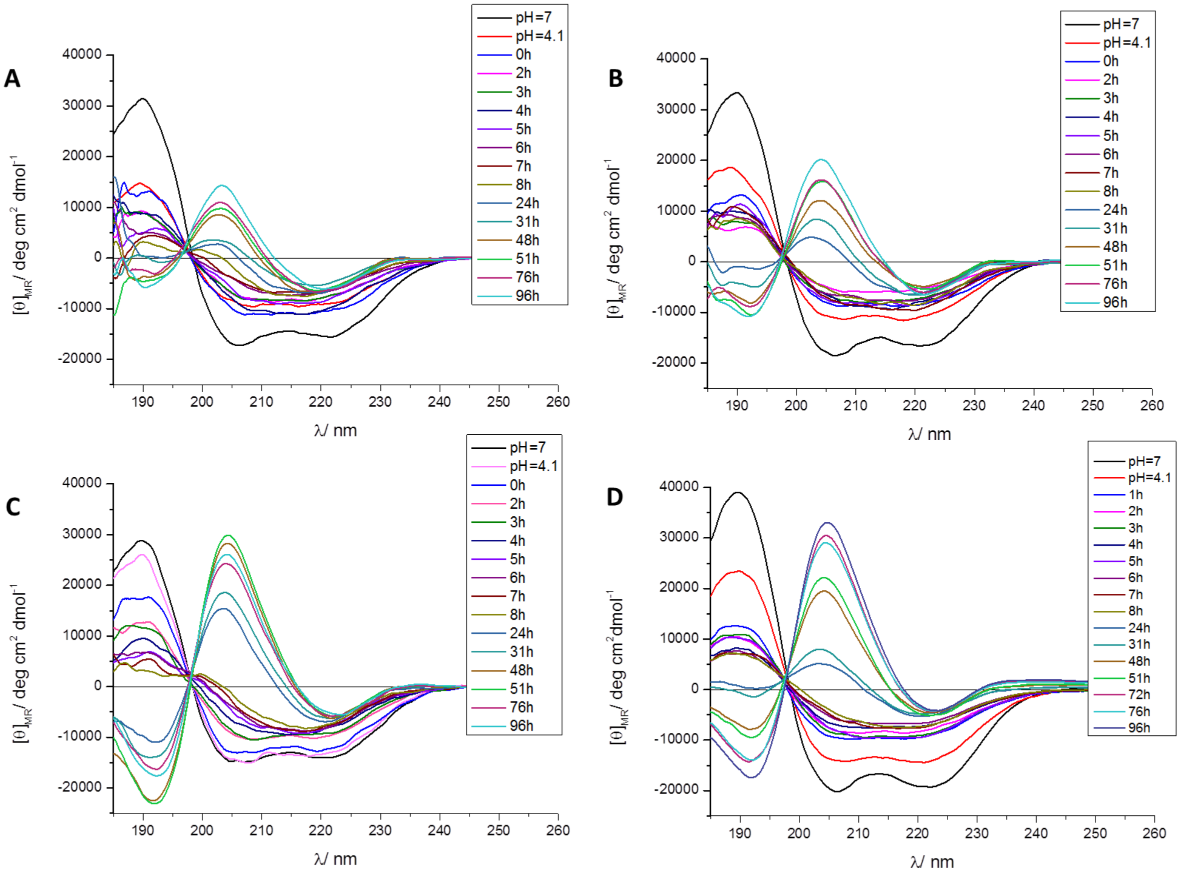

SFigure 10. Conformational changes determined by FUV-ECD as function of the time during amyloid formation at c_{E5}= 400 µM, c_{NaCl}= 50 mM (**A**) and c_{E5}= 400 µM, c_{NaCl}= 25 mM, (**B**) and c_{E5}= 400 µM, c_{NaCl}= 12.5 mM (**C**) and c_{E5}= 400 µM, c_{NaCl}= 0 mM (**D**) at *pH*= 4.1, stirring at *T*= 37 °C.

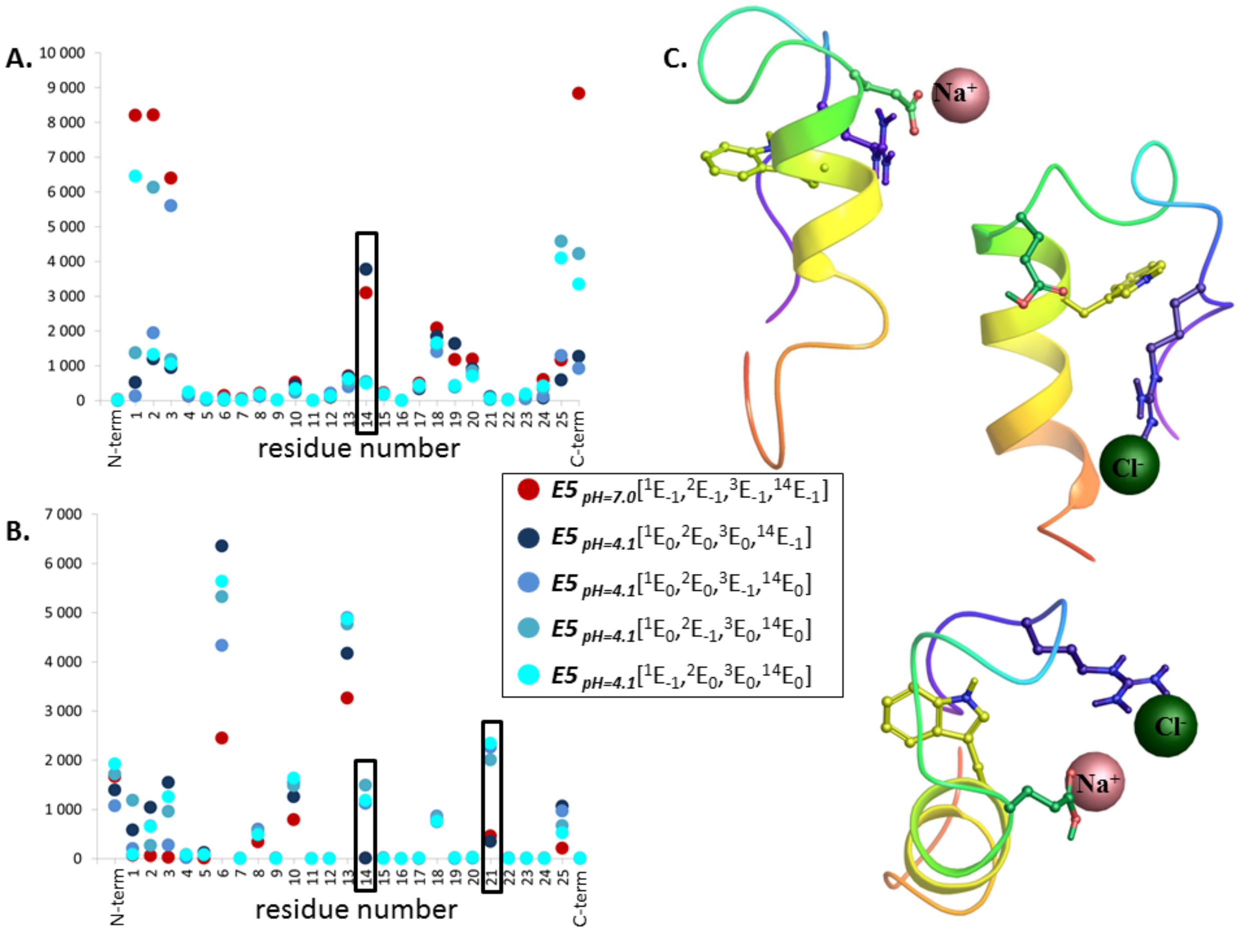

SFigure 11. Spatial distribution of Na^+ and Cl^- ions near (<3.5 Å) the heteroatoms of E5. The number of Na^+ (**A**) and Cl^- (**B**) ions found within interacting distance of the individual residues during the last 500 ns (125000 snapshots) of the MD simulations of the monomeric E5 at different *pH* dependent protonation microstates. **C)** Examples of ion-protein interactions selected from the MD simulations.

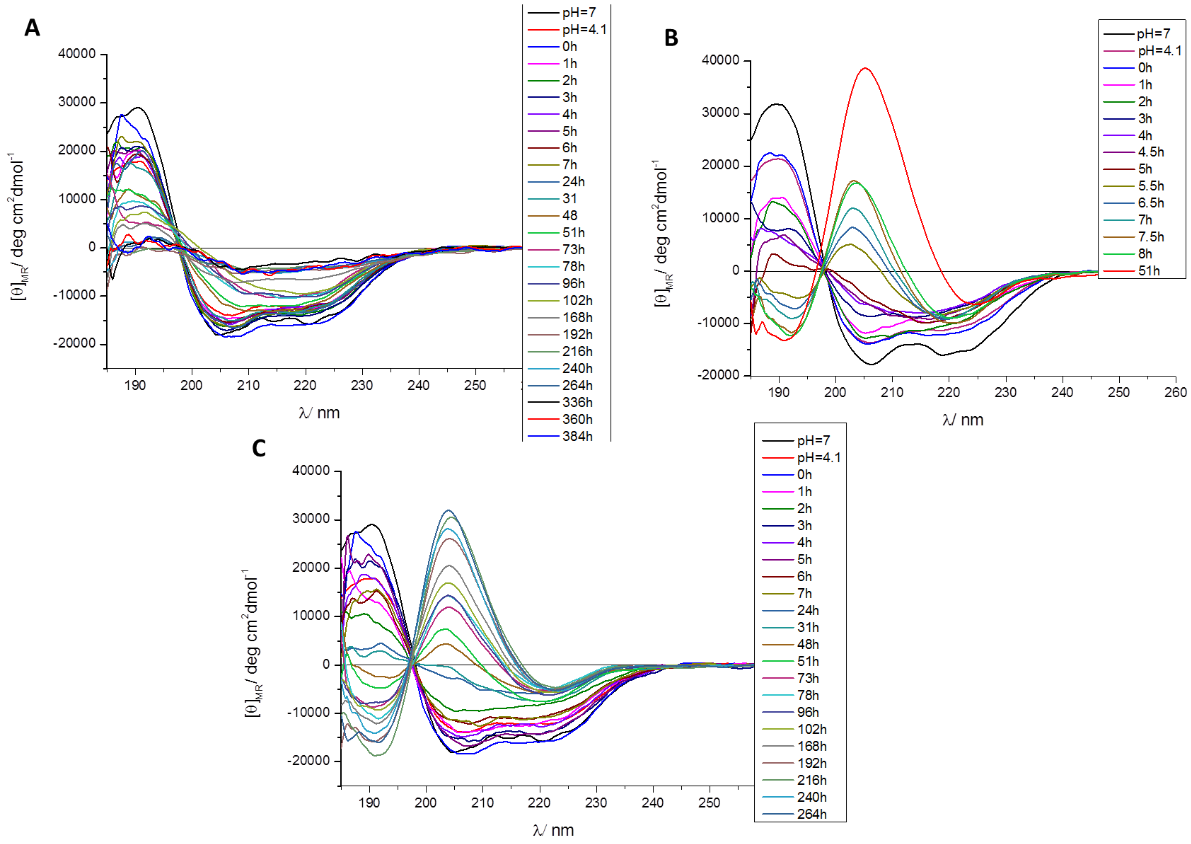

SFigure 12. Conformational changes determined by FUV-ECD as function of the time during amyloid formation at c_{E5}= 250 µM, c_{NaCl}= 50 mM, pH= 4.1 stirring at T= 23 °C (**A**) and at T= 37 °C, (**B**) at T= 47 °C (**C**).

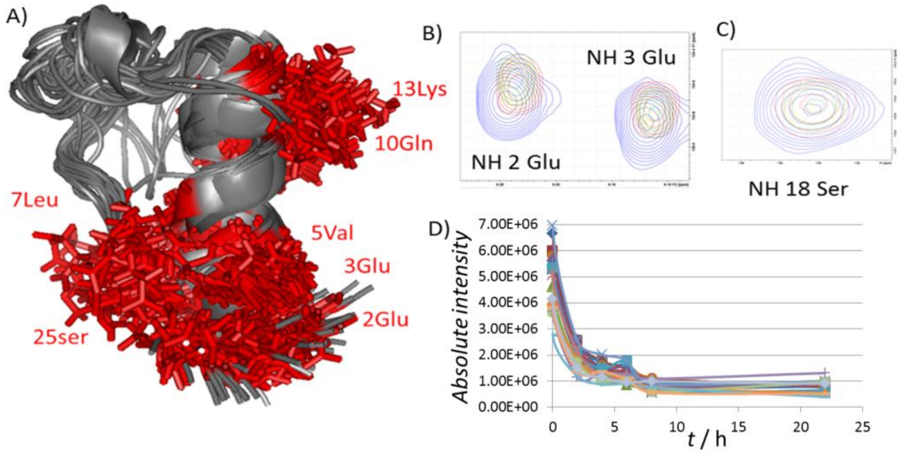

SFigure 13. A) The superimposed best 50 structures of E5. NHs of Glu^{2}, Glu^{3}, Val^{5}, Leu^{7}, Gln^{10}, Lys^{13} and Ser^{25} (highlighted red) shift the most during amyloid forming (**STable 2**). **B)** Selected ^{1}H-^{15}N-HSQC resonances of E5 shifting noticeably: t_{end} - t_0> 0.047 ppm. **C)** NH of Ser^{18} shifting none was used as a reference. **D)** The absolute intensity of the ^{1}H-^{15}N-HSQC resonances decay with time, indicating the loss of monomers and shorter oligomeric assemblies.

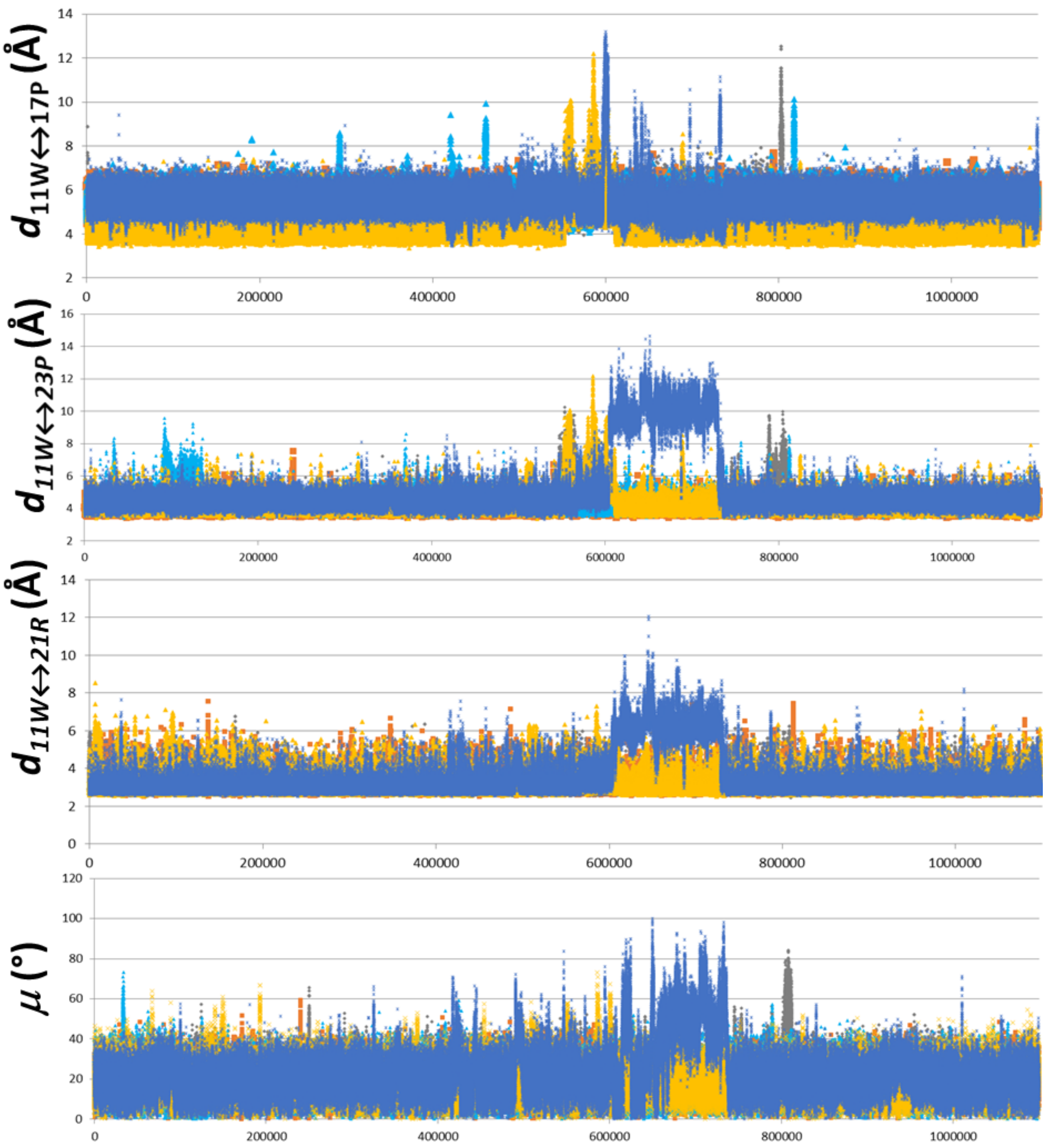

SFigure 14. Conformational measures signaling the degree of misfolding of E5 as function of the time for each trajectory. Grey indicates the measures of **E5**$[^{1}E_{-1}, {}^{2}E_{-1}, {}^{3}E_{-1}, {}^{14}E_{-1}]$ microstate, orange stands for **E5**$[^{1}E_{0}, {}^{2}E_{0}, {}^{3}E_{0}, {}^{14}E_{-1}]$; light blue for **E5**$[^{1}E_{0}, {}^{2}E_{0}, {}^{3}E_{-1}, {}^{14}E_{0}]$; yellow for **E5**$[^{1}E_{0}, {}^{2}E_{-1}, {}^{3}E_{0}, {}^{14}E_{0}]$ and deep blue for **E5**$[^{1}E_{-1}, {}^{2}E_{0}, {}^{3}E_{0}, {}^{14}E_{0}]$. Between 600-800 ns time window of the trajectory a significant change is reported by the measures in the single case of **E5**$[^{1}E_{-1}, {}^{2}E_{0}, {}^{3}E_{0}, {}^{14}E_{0}]$, namely the opening event of the Trp-cage.

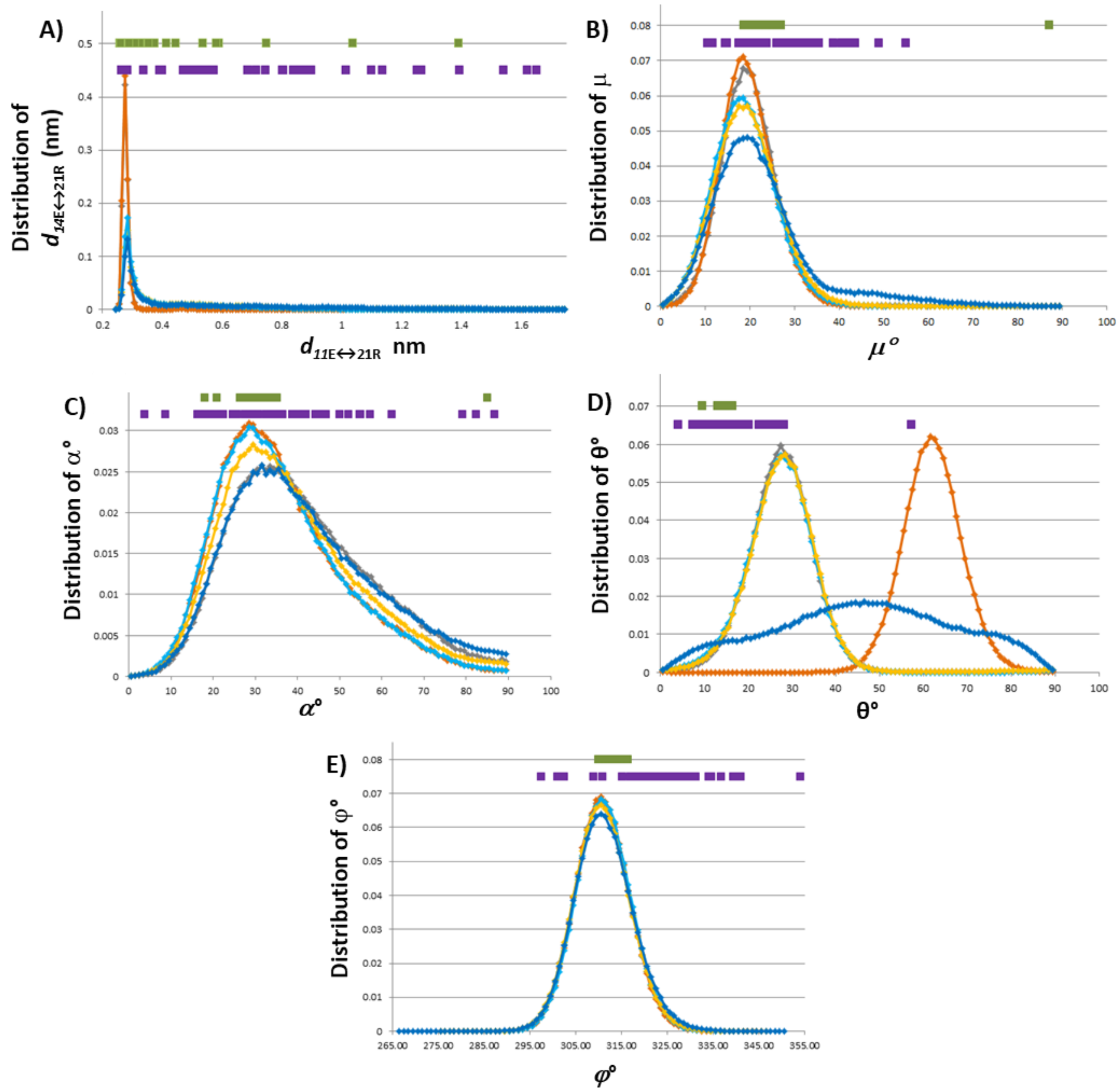

SFigure 15. Additional distributions of conformational measures are selected to signal the degree of misfolding of E5. **A-E)** The distribution of the selected measures for the MD trajectories (grey – *pH*= 7, **E5**[$^{1}E_{-1}$,$^{2}E_{-1}$,$^{3}E_{-1}$,$^{14}E_{-1}$] ; orange – *pH*= 4.1 **E5**[$^{1}E_{0}$,$^{2}E_{0}$,$^{3}E_{0}$,$^{14}E_{-1}$] ; light blue – *pH*= 4.1 **E5**[$^{1}E_{0}$,$^{2}E_{0}$,$^{3}E_{-1}$,$^{14}E_{0}$]; yellow – *pH*= 4.1 **E5**[$^{1}E_{0}$,$^{2}E_{-1}$,$^{3}E_{0}$,$^{14}E_{0}$]; blue – *pH*= 4.1 **E5**[$^{1}E_{-1}$,$^{2}E_{0}$,$^{3}E_{0}$,$^{14}E_{0}$] are plotted, while the distribution of the selected measures for NMR structures (green – *pH*= 7, purple – *pH*= 4.1) are indicated for every single structures above the MD distribution curves in a linear 1D manner. Measures of A) and B) characterize the tertiary structure of the Trp-cage, while measures of C, D, E describe the spatial arrangement of the aromatic rings (Trp^{8}, Trp^{11}).

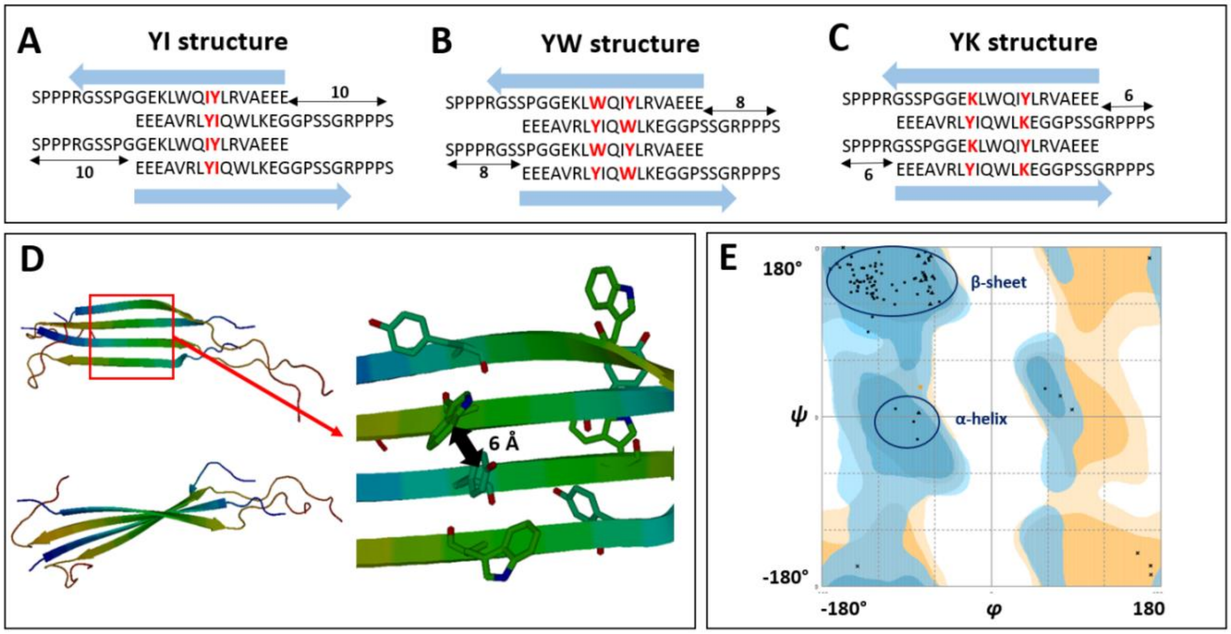

SFigure 16. A-C) The three different β-strand offsets of $(E5)_4$ used for MD simulations. In each of the composed models, the central helix was extended and Tyr^8 was paired antiparallel either with Ile^9, Trp^{11} or Lys^{13} residues of the adjacent β-strand. **D**) The most populated amyloid-like tetramer cluster of YW (*pH*= 4.1 between 100-340 ns) have shifted face-to-face π-π aromatic ring orientations (with ring separations of ~6 Å). On the contrary, neither YI, nor YK offsets made stable enough tetramers. **E)** The Ramachandran plot of the most populated cluster of the $(E5)_4$ YW structure with residues mainly located in the β-valley.

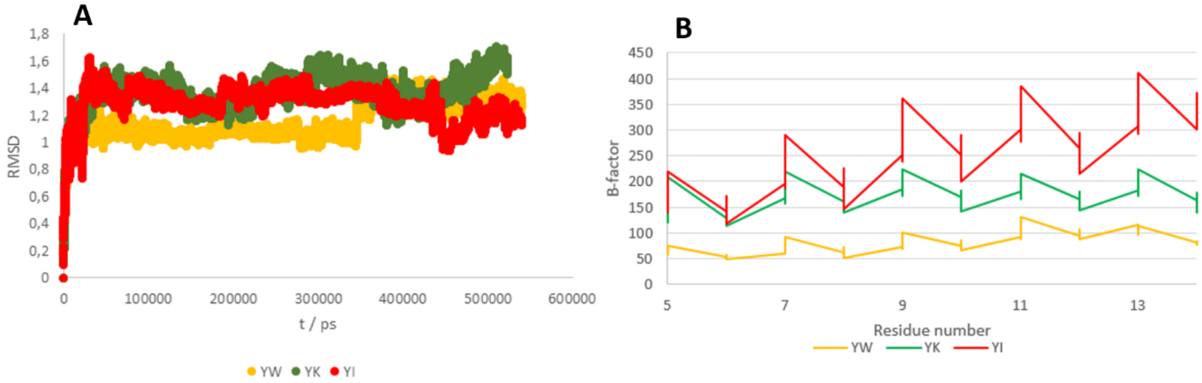

SFigure 17. RMSD values of the backbone of the full sequence (**A**) and B-factors of the backbone of the core region (V^5-K^{13}) of the two middle chains (**B**) of the tested offsets (YW, YK and YI) at *pH*= 4.1. The YW offset has the most stable backbone at region V^5-K^{13}, which is the aggregation core.

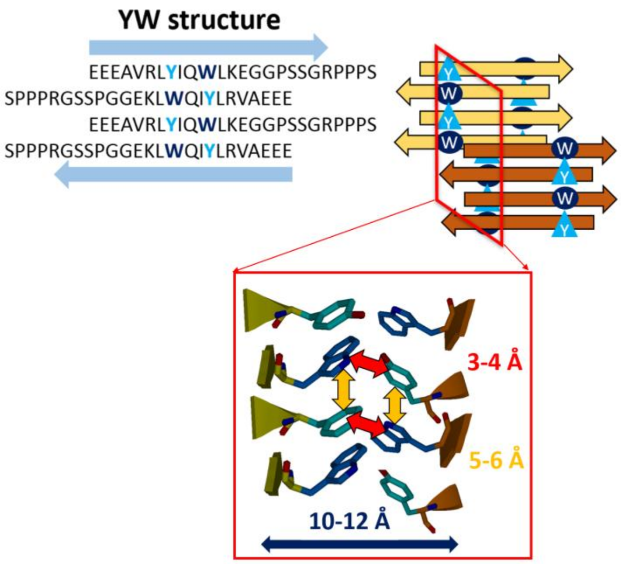

SFigure 18. Schematic illustration of a hypothetic late phase intersheet orientation of the aromatic rings (Tyr: light blue, Trp: dark blue). The intrasheet distance between the center of masses of the two aromatic side chains is 5-6 Å (yellow double arrows), while possible intersheet aromatic-aromatic distance can be 3-4 Å (red double arrows), while the sheet-sheet distance is 10-12 Å (blue double arrow).

STable 1.: *Conditions applied for ^{1}H-^{1}H-NOESY experiments (the resolution was always 2k x 512), and selected structural results retrieved.*

T / K	*pH*	Concentration	ns	Assigned crosspeaks	$\Sigma NOE^{i \rightarrow (i+5<)}$	Backbone all atoms RMSD (Å)
277	7	1.295mg/600µl	32	666	90	0.705
288	7	1.295mg/600µl	32	590	77	0.475
299	7	1.295mg/600µl	32	522	52	0.893
310	7	1.295mg/600µl	32	458	46	0.640
321	7	1.295mg/600µl	32	221	16	3.401
288	6	1.989mg/~800µl	56	550	64	0.732
288	5	1.989mg/~800µl	48	319	16	2.564
288	4	0.842mg/600µl	128 cryo	339	22	1.833
288	2	1.989mg/~800µl	96	748	84	0.516

STable 2. Combined ^{1}H- and ^{15}N-chemical shift changes, $\Delta(t_{end})$- $\Delta(t_0)$, of E5 recorded for the first 24h of the amyloid formation[a]

	Glu^{1}	Glu^{2}	Glu^{3}	Ala^{4}	Val^{5}
$\Delta(t_{end})$ - $\Delta(t_0)$	n.d.	0.139[b]	0.081	0.013	0.108
residue in E5	Arg^{6}	Leu^{7}	Tyr^{8}	Ile^{9}	Gln^{10c}
$\Delta(t_{end})$ - $\Delta(t_0)$	0.011	0.055	0.014	0.047	0.079
residue in E5	Trp^{11}	Leu^{12}	Lys^{13}	Glu^{14}	Gly^{15}
$\Delta(t_{end})$ - $\Delta(t_0)$	0.003	0.036	0.058	0.024	0.023
residue in E5	Gly^{16}	Pro^{17}	Ser^{18}	Ser^{19}	Gly^{20}
$\Delta(t_{end})$ - $\Delta(t_0)$	0.029	n.d.	0.020	0.002	0.030
residue in E5	Arg^{21}	Pro^{22}	Pro^{23}	Pro^{24}	Ser^{25}
$\Delta(t_{end})$ - $\Delta(t_0)$	0.003	n.d.	n.d.	n.d.	0.175

[a] *pH*= 4.1, *T*= 37 °C, c_{E5}= 600 µM, c_{NaCl}= 12.5 mM, ζ= stirring

[b] All changes, $\Delta(t_{end})$- $\Delta(t_0)$, larger than the average, <Δ> = 0.047 are highlighted red

[c] Gln side chain amides shift <0.103> ppm

References:

1 Vranken, W. F.; Boucher, W.; Stevens, T. J.; Fogh, R. H.; Pajon, A.; Llinas, M.; Ulrich, E. L.; Markley, J. L.; Ionides, J.; Laue, E.D., The CCPN data model for NMR spectroscopy: development of a software pipeline. *Proteins* **2005**, 59(4), 687-96.

2 Rieping, W.; Habeck, M.; Bardiaux, B.; Bernard, A.; Malliavin, T. E.; Nilges, M., ARIA2: automated NOE assignment and data integration in NMR structure calculation. Bioinformatics **2007**, 23(3), 381-2.

3 Brunger, A. T., Version 1.2 of the Crystallography and NMR system. *Nature Protocols* **2007**, 2, 2728–2733.

4 Kozakov, D.; Brenke, R.; Comeau, S.R.; Vajda, S. PIPER: An FFT-based protein docking program with pairwise potentials. *Proteins: Structure, Function, and Bioinformatics*. **2006**, 65(2):392-406.
